# Nanodiscoidal Nucleic Acids for Gene Regulation

**DOI:** 10.1021/acschembio.3c00038

**Published:** 2023-11-01

**Authors:** Radhika Sharma, Steven Narum, Shuhong Liu, Yixiao Dong, Kyung In Baek, Hanjoong Jo, Khalid Salaita

**Affiliations:** † Department of Chemistry, Emory University, Atlanta, Georgia 30332, United States; § Wallace H. Coulter Department of Biomedical Engineering, Georgia Institute of Technology and Emory University, Atlanta, Georgia 30332, United States

## Abstract

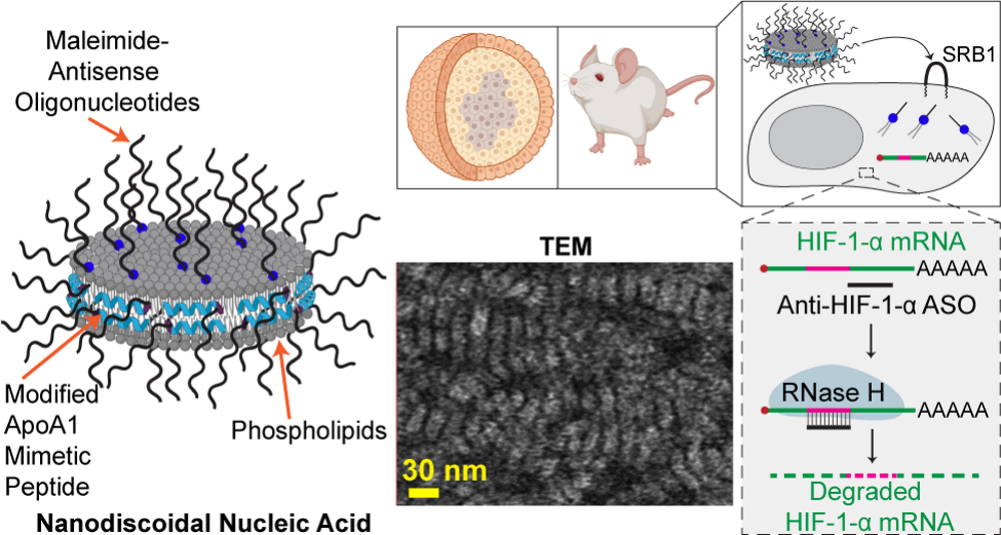

Therapeutic nucleic acids represent a powerful class of drug molecules to control gene expression and protein synthesis. A major challenge in this field is that soluble oligonucleotides have limited serum stability, and the majority of nucleic acids that enter the cells are trapped within endosomes. Delivery efficiency can be improved using lipid scaffolds. One such example is the nanodisc (ND), a self-assembled nanostructure composed of phospholipids and peptides and modeled after high density lipoproteins (HDLs). Herein, we describe the development of the nanodiscoidal nucleic acid (NNA) which is a ND covalently modified with nucleic acids on the top and bottom lipid faces as well as the lateral peptide belt. The 13 nm ND was doped with thiolated phospholipids and thiol-containing peptides and coupled in a one-pot reaction with oligonucleotides to achieve ∼30 DNA/NNA nucleic acid density. NNAs showed superior nuclease resistance and enhanced cellular uptake that was mediated through the scavenger receptor B1. Time-dependent Förster resonance energy transfer (FRET) analysis of internalized NNA confirmed that NNAs display increased stability. NNAs modified with clinically validated antisense oligonucleotides (ASOs) that target hypoxia inducible factor 1-α (HIF-1-α) mRNA showed enhanced activity compared with that of the soluble DNA across multiple cell lines as well as a 3D cancer spheroid model. Lastly, *in vivo* experiments show that ASO-modified NNAs are primarily localized into livers and kidneys, and NNAs were potent in downregulating HIF-1-α using 5-fold lower doses than previously reported. Collectively, our results highlight the therapeutic potential for NNAs.

## Introduction

Nucleic acid therapeutics have made rapid advancements and strides over the past couple decades, which were only accelerated during the COVID-19 pandemic because of the role of self-assembled lipid nanoparticle-mRNA vaccines in combatting severe disease. The numerous clinical trials using nucleic acid-based drugs including antisense oligonucleotides (ASOs), siRNA, mRNA, adenoviral vectors (AAVs), and aptamers are starting to yield a steady stream of FDA-approved therapeutics. That said, there are a significant number of failures, and hundreds of clinical trials thus far have only produced 15 FDA approved therapies to date. The efficacy of these drugs is often limited by several factors. First, the vast majority of nucleic acid drugs fail to reach their cytoplasmic target due to many processes that include nuclease-mediated degradation, as well as endosomal entrapment and degradation.^1,2^ To overcome this issue, larger doses of nucleic acid drugs are used, which can lead to thrombocytopenia and off-target effects.^3^ Nanoparticle (NP) compositions, such as spherical nucleic acids, help mitigate the delivery and stability challenges and have been shown to improve specificity, therapeutic dosing, and overall half-life.^4,5^ In part, NPs are an advantageous platform for delivery due to their smaller sizes (<200 nm) which leads to enhanced cell uptake and evasion of clearance processes. Phospholipid-based NPs (e.g., liposomes) are the most widely used NP scaffold in the clinic, however, conventional liposomes tend to have limited stability and lower encapsulation efficiencies.^6^ Accordingly, lipid-based nanodiscs (NDs) have emerged as a promising class of NPs for the delivery of nucleic acids.^7,8^ NDs resemble pre-ß high-density lipoproteins (HDLs) and are discoidal-shaped nanostructures (dimensions: ∼10 nm wide × 5 nm thick), and this nonspherical shape and its ultrasmall size can be particularly advantageous for drug delivery because it offers low strain energy during cell uptake, compared to nanoparticles of other geometries and sizes.^9^

NDs structurally mimic nascent HDL that circulate in blood and function in reverse cholesterol transport.^10^ Due to this native role, NDs are endowed with anti-inflammatory^11^ and antiatherogenic properties,^12^ hence offering an additional benefit in using the ND scaffold. Additionally, NDs are also natively loaded with micro-RNAs (<25 nucleotide long RNAs) through charge complexes between the RNA and the lipids and play an important role in regulating HDL metabolism and other tissue-signaling processes.^13,14^ NDs are primarily composed of phospholipids along with apolipoprotein A1 (ApoA1), an α-helical and amphipathic scaffolding protein. ApoA1 interacts with scavenger receptor B1 (SRB1) protein expressed on cell surfaces to mediate the influx and efflux of cholesterol and miRNA cargo from the cytosol of the cell. This SRB1-ND transport process is described as nonendocytic^15^ and has been shown to enhance the delivery of oligonucleotides from the ND surface to the cell because this transport mechanism bypasses endosomal entrapment and degradation.^14,16,17^ SRB1 is expressed in multiple cell types and especially in cancer cells, hence offering a broad strategy for targeted delivery of oligonucleotides.^18−20^ Moreover, NDs can greatly improve circulation times and avoid immune-triggered elimination which plagues the delivery of conventional oligonucleotides. Furthermore, NDs can be rapidly self-assembled from mixtures of phospholipids and ApoA1 (<3 h), offer facile scale-up, are regarded as safe, and have demonstrated high tolerability in previous clinical trials.^21^ Therefore, developing bioconjugation strategies for linking the maximum number of nucleic acids to each ND is paramount and may transform current approaches for nucleic acid-based drugs.^5,22^

Previously, we reported a method to boost nucleic acid density on a ND scaffold by doping in thiol-containing phospholipids into the ND and conjugating these lipids to maleimide-modified oligonucleotides and forming covalent linkages.^23^ This conjugation chemistry offered significant advantages over commonly used noncovalent modifications of spherical and discoidal HDL scaffolds (including HDL-mimicking peptide phospholipid scaffold (HPPS), synthetic high-density lipoprotein (sHDL), and NDs).^1,24−26^ Specifically, noncovalent interactions employing electrostatic binding and cholesterol-mediated binding are weak, yield low nucleic acid density (∼1–10 per ND)^7,17,27,28^ and high polydispersity of loading, and display short half-lives (2–4 h) *in vitro* and *in vivo*,^16,29^ thus limiting translational potential. In this work, we sought to engineer the ND scaffold to maximize the loading density on the ND scaffold. The premise for this approach is the rigor of prior work with spherical nucleic acids that showed that high density DNA loading boosts the efficacy of these therapeutics through several mechanisms including nuclease suppression, enhanced uptake, and increased hybridization efficiency to mRNA targets.^22,30^

Given that >50% of the ND is composed of the peptide scaffold, we postulated that we could increase DNA loading by directly linking the nucleic acid to the peptide spanning the perimeter of the ND. The challenge with this approach is that it requires introduction of a reactive group to the amphipathic peptide which may disrupt its propensity to form ND because subtle and in some cases, single amino acid modifications have been shown to abolish its activity.^31^ Therefore, we screened a small library of cysteine-modified ApoA1-mimetic peptides focusing on a 22 amino acid (22A) target that had been tested in phase I clinical trials to treat atherosclerosis.^32^ We decided to use reactive thiol residues because this is a native amino acid which is likely less immunogenic, and given that we can introduce thiol-containing phospholipids into the ND, having thiolated peptides suggests the potential for running an efficient one-pot reaction to couple oligonucleotides to ND using facile Michael addition chemistry. In this way, we aimed to create a nanodiscoidal nucleic acid (NNA) with DNA loaded on the perimeter as well as on the top and bottom lipid faces of the structure. We inserted cysteines (Cys’s) on the N-, C-, or both termini of the peptide and identified peptides that efficiently formed homogeneous populations of NNAs. The optimal NNAs employed C-terminal GGC-modified residues and presented an average of 30 copies of DNA per NNA, the highest density of nucleic acid–ND conjugates reported to date. The dense NNA structure retained its ultrasmall size (∼12 nm) and discoidal morphology and demonstrated significant nuclease and serum stability. By conjugating the clinically relevant EZN2968, an antisense oligonucleotide (ASO) that targets hypoxia inducible factor 1-α (HIF-1-α) mRNA to the ND,^33^ we demonstrated avid *in vitro* uptake along with reduction in HIF-1-α transcript levels and a marked decrease in cell viability of cancer cells that are HIF-1-α dependent. We used this specific ASO because of the role of HIF-1-α in promoting the survival of cancerous and tumorous tissue. This ASO showed activity in phase I clinical trials against refractory solid tumors, but this was not pursued further, suggesting that enhancing its efficacy may offer a route to rescuing this candidate. We further confirmed that the NNA uptake is SRB1 dependent. The efficacy of anti-HIF-1-α NNAs was further validated using 3D spheroid models that showed enhanced uptake that was SRB1-mediated with an ∼2-fold enhancement in transcript knockdown compared to identical concentrations of naked ASO. Lastly, we confirmed the delivery and activity of anti-HIF-1-α NNA conjugates *in vivo* and specifically in liver and kidney tissues using a murine model. Importantly, we observed activity at low dosing (0.7 mg/kg) which afforded a 5-fold enhancement compared to the conventional ASO that dosed multiple times.^33^ Overall, this work demonstrates marked improvements in the efficacy of the HIF-1-α ASO, which has been tested in human trials, and thus suggests the potential for NNAs to transform the efficacy of ASO drugs broadly.

## Results and Discussion

### Screening of Cysteine-Modified ApoA1 Mimetic Peptides

**Figure 1:**
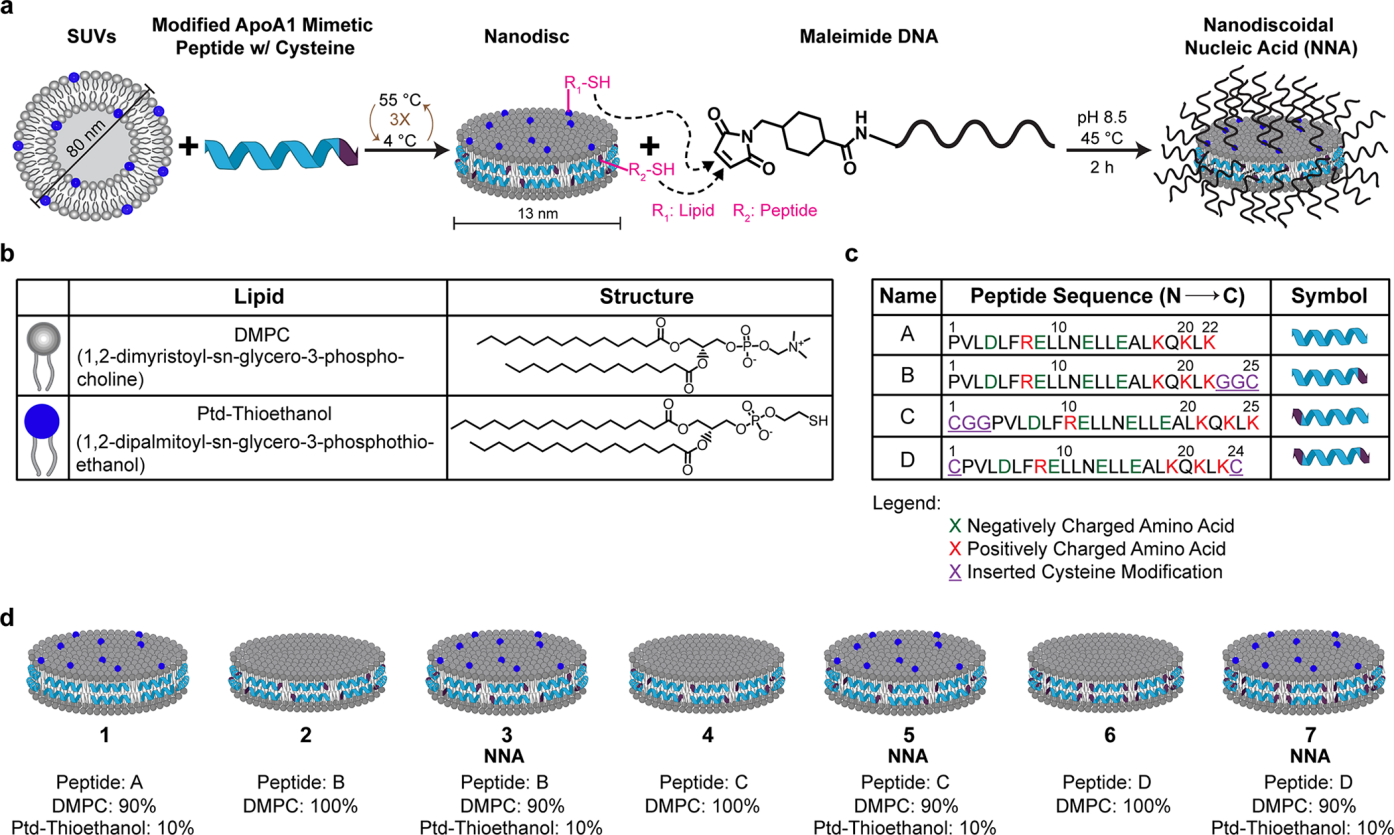
Assembly and synthesis of nanodiscoidal nucleic acids (NNAs). (a) Scheme depicting the assembly of NNAs. The ND scaffold is assembled by preparing 80 nm small unilamellar vesicles (SUVs) and combining them with a modified ApoA1 mimetic peptide containing a Cys amino acid insertion. The NNA is generated by conjugating maleimide-linked DNA to the exposed thiols on the surface (lipid) and edge (peptide) of the scaffold. (b) Table of lipids used in the assembly of NDs and NNAs. DMPC is the majority component present in the NDs and the thiol lipid, Ptd-thioethanol (blue), is added to certain discs to prepare NNAs. (c) Table of ApoA1 mimetic peptides screened for the formation of NNAs. The original mimetic peptide, denoted as A, does not contain any Cys residues and was further modified in versions B–D at the N- and/or C-terminus. (d) ND scaffolds generated from the peptide screen as shown in (b). Excluding peptide A, each peptide was used to prepare two different versions of DNA conjugates, one with DMPC exclusively and referred to as NDs and a second type which included thiolated phospholipids and denoted as the NNA.

ND scaffolds were assembled by preparing small unilamellar vesicles (SUVs) through extrusion (Figure 1a).^23^ SUVs were composed of phospholipids, specifically 1,2-dimyristoyl-*sn*-glycero-3-phosphocholine (DMPC) and/or 1,2-dimyristoyl-*sn*-glycero-3-phosphothioethanol (Ptd-thioethanol) in a 90:10 (% molar) ratio (Figure 1b). Cy5 headgroup tagged phospholipid (Table S2) was added at a 0.15–1.0% molar ratio as needed to visualize NDs using fluorescence. We chose to work with an ApoA1 mimetic sequence, 22A, that has been clinically evaluated in a phase I safety analysis and demonstrated to be tolerable at high doses.^32^ The NNA scaffolds were created by inserting one or two Cys residues at the N- and/or C- terminus of the 22A peptide (peptide A, Figure 1c) to generate NDs with peptides B, C, and D. To conjugate nucleic acids to the top and bottom faces of the ND, we doped the ND with 10% thiol-modified phospholipids to enable nucleic acid conjugation to the phospholipids. This lipid composition was used in our experiments because this was previously identified as the optimal doping ratio of thiol lipids to form NDs without agitating the morphology and DNA density from pronounced disulfide bonds that maybe present at higher thiol lipid concentrations.^23^ The peptides containing one Cys insertion at the N- or C-terminus (peptides B and C) also included a double glycine spacer to minimize disruption to the native amphipathic structure of the peptide. The double-Cys insertion (peptide D) into 22A peptide did not contain a double glycine spacer as the helical wheel modeling of the structure suggested that adding 6 residues would perturb the class A helix structure of the peptide.^34^ The sequence of ApoA1 mimetic peptides, while bearing no homology to native ApoA1, is strategically designed to mimic the alpha helical design of its native counterpart. These peptides are typically composed of specific polar and nonpolar amino acids that are strategically positioned within the sequence, which endows the sequence with a similar structure to ApoA1. We carefully examined the helical wheel diagrams (Figure S1) to ensure that our designs did not disturb the class A helical structure, thereby minimizing potential for the modified peptides to disrupt discoidal complexes and/or impact SRB1 recognition. The peptides were capped with an acetyl group at the N-terminus and an amide group at the C-terminus for each of these peptides to boost lipid affinity.^35^ Following the ND formation, maleimide-modified DNA was coupled to the scaffold to create DNA–ND and NNA conjugates (Figure 1d) using optimized reaction conditions identified previously to bolster reaction rate and nucleophilicity.^23^ In this work, we reserve the notation NNA for NDs that present nucleic acids on all faces of the structure, both the phospholipid headgroups as well as the peptide perimeter using the ND scaffolds 3, 5, and 7 as shown in Figure 1d. We refer to structures that present nucleic acids solely on phospholipids or solely on peptides as DNA–ND conjugates.

**Figure 2:**
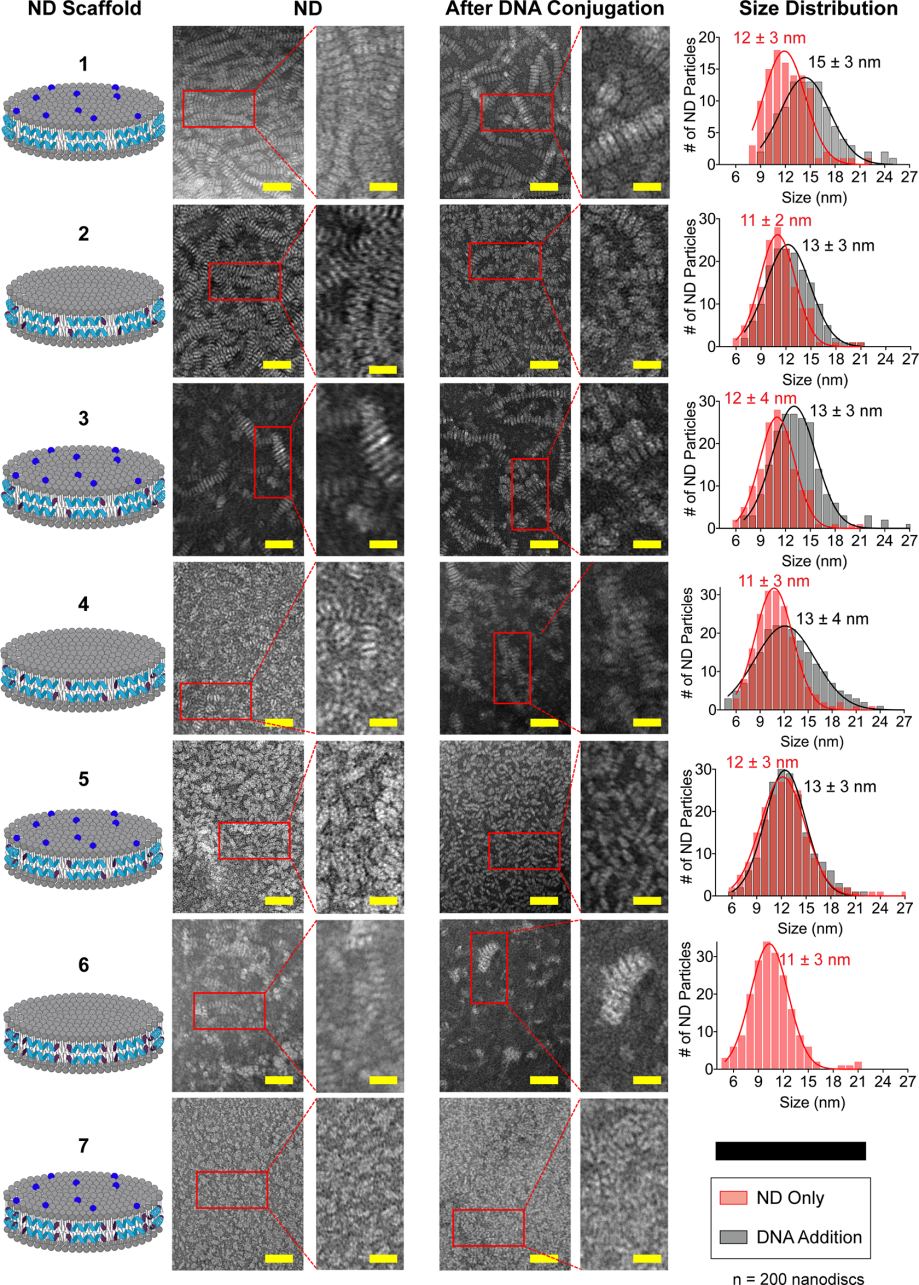
Transmission electron microscopy imaging of NDs and NNAs. Representative TEM images were shown for each ND prior to and after DNA conjugation. Samples were prepared using a plasmon-etched 400-mesh copper grid and negatively stained using Nano-W. Scale bar: 50 nm; inset: 20 nm. Histograms represent binned diameter size distribution of NDs (*n* = 200) before (red) and after (black) DNA coupling for each scaffold (1–6). ND 6 after DNA conjugation and ND 7 before and after DNA conjugation appeared to form lipid aggregates instead of discoidal NDs, and therefore, we do not show histograms for these NDs.

The DNA–ND and NNA conjugates were visualized before and after DNA coupling by using transmission electron microscopy (TEM) (Figure 2). The imaging revealed a monodisperse, homogeneous morphology with “coin-like” stack formations, attributed to the rouleaux effect from the negative staining process,^36,37^ for NDs 1–5 before and after conjugation to DNA (Figure 2). Furthermore, stacking behavior could be driven by the dehydration of the ND during the sample preparation process and can promote a stacking orientation.^38^ In contrast, NDs assembled using peptide D (Figure 1c) showed heterogeneous morphology prior to and after DNA conjugation. The only exception was ND 6 that showed some stack formation prior to DNA conjugation but these were more disorganized than other NDs tested (1–5). The weaker propensity to form intact ND for 6 and 7 is potentially due to the N- and C-Cys-modified termini which increase the probability of forming disulfide bridges and aggregation. For NDs 1–5, there was a small (nonsignificant) increase in diameter as measured by TEM, which showed that ND size changing from ∼11–12 nm before DNA conjugation to ∼13–15 nm after coupling. Dynamic light scattering (DLS) measurements (Figure S2) showed a much more substantial increase in particle size as the hydrodynamic radius shifted from ∼10–13 nm before DNA addition to ∼14–22 nm after DNA addition. This larger change in size observed in DLS is consistent with the literature for gold nanoparticle and lipid nanoparticles after conjugation with DNA.^39,40^ Therefore, TEM and DLS confirm the ultrasmall size of the native scaffold following DNA coupling.

Interestingly, the intensity-normalized DLS data show an increase in the polydispersity of NDs and specifically the appearance of larger-diameter particles, which was most pronounced for ND 5 (Figure S2). This is due to the formation of a small population of liposomal aggregates that likely form due to formation of disulfide bridges between ND during the DNA coupling reaction as well as destabilization of the ND following DNA coupling. These aggregates were more distinct for NNA (ND 5) rather than the peptide–DNA and phospholipid–DNA conjugate ND suggesting: (1) that high density DNA on the ND can lead to slight destabilization of the ND and (2) that introducing the Cys to the N-terminus of 22A was slightly more destabilizing. Helical wheel projections indicated that the peptide C places the N-terminal Cys, which is considered polar, within the hydrophobic face of the peptide and may explain the decreased stability of ND 4 and 5 compared to that of ND 2 and 3 (Figure S1). It has been previously noted that the ND samples display a minor fraction of larger liposomal aggregates and those aggregates scatter light strongly compared to the smaller NDs, hence amplifying the signal in intensity-based DLS measurements.^41^ This is consistent with our observation as we were not able to see these subpopulations of liposomal aggregates in TEM and confirms that these species are minor. Taken together, this screen of Cys-modified 22A peptides and thiol containing phospholipids confirms that we generated NNAs that are monodisperse maintaining an ∼5 nm × ∼13 nm disc-like structure based on TEM.

**Figure 3:**
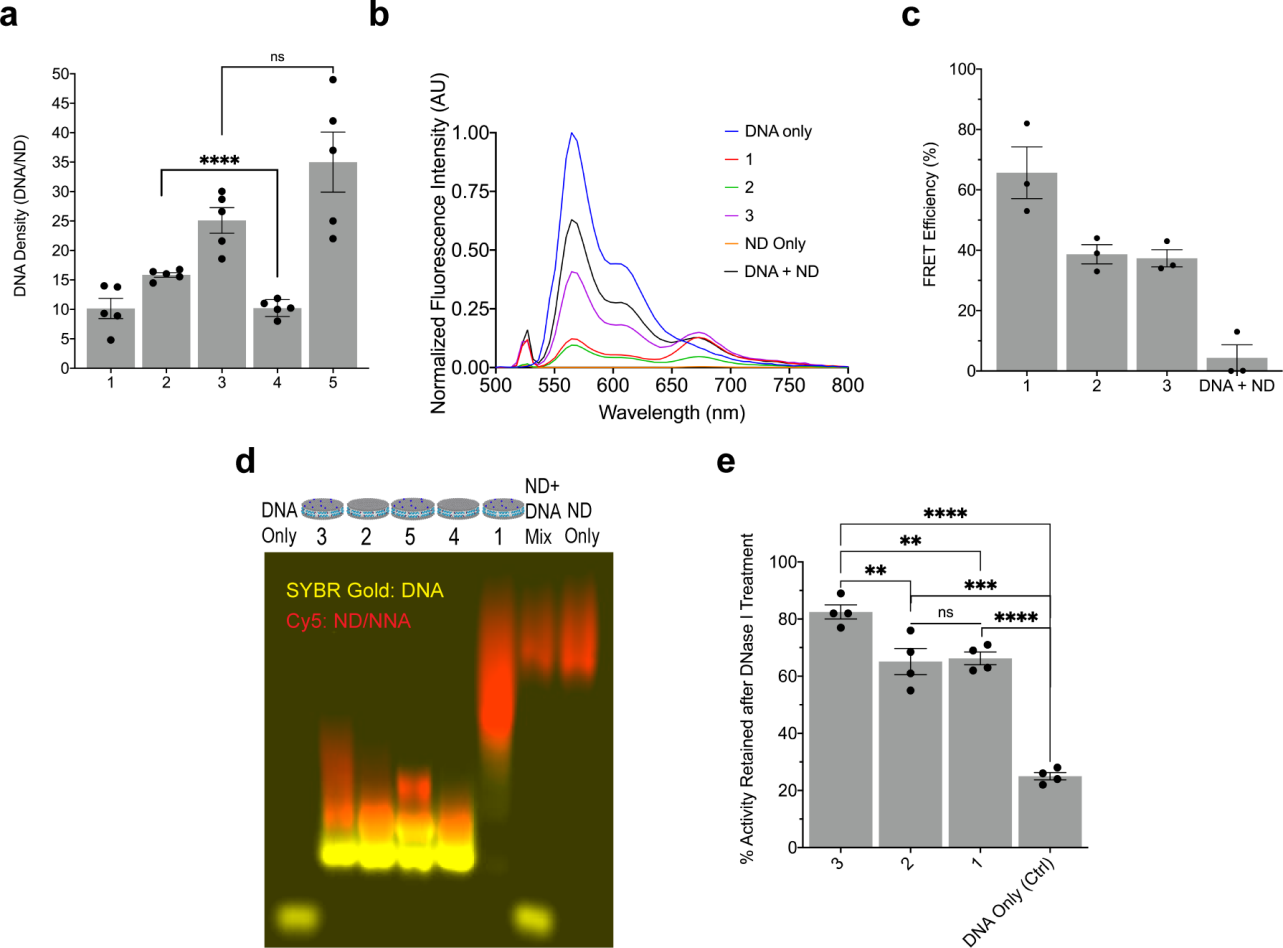
Characterization of DNA–ND and NNA conjugates and their nuclease resistance. (a) DNA density plot comparing the number of DNA strands per ND scaffold (1–5) after covalently conjugating maleimide–DNA to the thiol/cysteine bearing NDs. There is an increase in the amount of DNA added to an ND when using the NNA scaffolds (NDs 3 and 5). Data represent an average of *n* = 5 independent replicates. (b) Fluorescence spectra for representative samples of DNA–NDs (NDs 1–2) and NNA (ND 3) prepared with 1% Cy5 phospholipid and DNA tagged with TYE563 fluorophore. Fluorescence spectra were collected for DNA (100 nM) concentration when the samples were excited at λ = 525 nm. (c) Plot of the calculated FRET efficiency for representative samples of DNA–NDs and NNAs against the DNA mixed with ND (no attachment). The higher FRET efficiencies (37–66%) present in the conjugated sample compared to the control (∼2%) validate the attachment of the DNA on the surface and the peptide of the ND. Data represent an average of *n* = 3 independent replicates. (d) Agarose gel electrophoresis of the DNA–ND and NNA conjugates (NDs 1–5), DNA mixed with ND, DNA only, and ND only samples. NDs (red) were prepared with 0.15% Cy5 phospholipid, and the gel for sample analysis was prepared with 1.5% agarose in TAE buffer with in-gel staining for DNA using SYBR gold (yellow). Retardation of the DNA bands (NDs 1–5) compared to the unbound free DNA control further indicates that the DNA is covalently bound to the discs. (e) Plot showing the percent activity retained after conjugating a catalytically active DNAzyme to the ND forming DNA–NDs (1–2) and NNA (3) and exposing the representative samples to 1 U of DNase I for 2 h. The NNA scaffold displayed enhanced protection against DNase I because it displayed higher activity (83%) compared to the DNA conjugated to the surface (1) and peptide (2), which retained ∼65% activity, whereas the unbound soluble DNAzyme (denoted as DNA) retained only 25% of activity. Each data point represents an independent replicate (*n* = 4). ** *p* < 0.01, *** *p* < 0.001, **** *p* < 0.0001. Error bars represent SEM.

We next measured DNA density on NDs 1–5 using the OliGreen assay, a commercial fluorescence assay that stains nucleic acids and can be used to measure total DNA present, and found that the NNAs (NDs 3 and 5) had the greatest DNA density per disc (Figure 3a, eqs 1–3). ND 1 only presented thiols on the phospholipid and led to 11 ± 5 DNA strands per ND. ND 2, which displayed Cys at the peptide C-terminus afforded 16 ± 1 DNA strands per ND. These values are consistent with our simple calculations^23,42^ that estimate that there are 4-fold more thiol groups on peptides compared to that of the phospholipids. Unsurprisingly, ND 3 displayed 25 ± 7 DNA strands per ND which suggests that DNA coupling can efficiently proceed on both the phospholipid and peptide with minimal steric clash. ND 4 displayed a lower density compared to the ND 2, and this is likely due to the stability issue of peptide D (Figure S1). Finally, ND 5 showed the largest density of 35 ± 14 DNA strands per ND. While the average density of these NNAs was the greatest, these values were heterogeneous and displayed high variability which likely relates to the broader size distributions measured by DLS. Accordingly, student’s *t* test analysis comparing the two NNAs did not show statistical significance in their DNA densities, and hence, ND 3 is more attractive as a therapeutic candidate given its enhanced monodispersity and consistent DNA density. We also compared the DNA density of NNAs to that of spherical nucleic acids composed of a gold nanoparticle core, which have similar diameters and present the highest density of DNA and showed that NNA are approaching the DNA densities for those structures (Table S1).

To validate that the DNA is covalently linked to the ND, we further performed a series of characterization experiments on representative DNA–ND and NNA samples using Förster resonance energy transfer (FRET) (Figure 3b,c). Here, FRET served as a spectroscopic ruler to validate the conjugation of DNA to the ND, as nanoscale proximity would lead to high FRET efficiency compared to our unlinked DNA which is expected to display minimal FRET. We used TYE563 fluorophore tagged ASOs that target HIF-1-α (Table S3) as the donor, whereas the ND was tagged with an acceptor dye (1% Cy5 headgroup-modified phospholipid). We excited the TYE563 donor at λ = 525 nm and collected the emission spectra (Figure 3b) quantifying sensitized emission from Cy5. Qualitatively, Cy5 emission at λ = 670 nm compared to direct donor emission (λ = 560 nm) was greatest for ND 1, 2, and 3 compared to controls where the DNA was not covalently linked to ND or when the ND lacked the Cy5 acceptor or when the donor was absent. FRET efficiency was quantified by using the ratio of donor emission normalized to the donor emission in the absence of the acceptor (eq 4), and this data is plotted in Figure 3c. Using this analysis, we found that the ND 1, 2, and 3 showed greater quantitative FRET (40–70%) compared to that of controls where the ND and DNA were present in the solution but not covalently linked (2%). The low FRET efficiency of the ND and DNA mixture compared to the higher FRET efficiency of the chemically bound DNA–ND and NNA data validates that DNA is chemically linked to the ND and is not physisorbed. Phospholipid-based nanomaterials primarily present zwitterionic phosphocholine headgroups which tend to minimize nonspecific binding of DNA.^43^ Interestingly, we also found that the ND with DNA linked to the peptide showed lower FRET efficiency compared with ND with DNA linkage to the phospholipid. This was consistent with the expected geometry of the ND with the peptide-linked DNA lying at the periphery of the ND and increasing the distance between the phospholipid headgroups with the acceptor and donor dyes.

To further validate the covalent attachment of the DNA to the ND, we next ran gel electrophoresis of NDs that were labeled with 0.15% Cy5 phospholipid and coupled to DNA. The gel is shown in Figure 3d, and for ease of visualization, SYBR Gold emission from the DNA was pseudocolored yellow, while the Cy5-phospholipid emission was pseudocolored red. NDs 1–5 that were conjugated to DNA showed different mobility compared to an ND scaffold only, DNA mixed with ND (but unconjugated), and soluble DNA only. As expected, upon DNA conjugation, the ND and associated phospholipid migrated more rapidly through the gel compared to the NDs lacking DNA. Additionally, the DNA mobility was slowed upon ND conjugation. Taken together, the gel electrophoresis results along with the FRET analysis confirm covalent coupling of the DNA to the ND both through peptide and phospholipid coupling.

Prior work on DNA-modified gold nanoparticles showed that increasing the density of DNA can lead to enhanced DNase resistance,^5,22^ and thus, we next tested whether NNA’s demonstrate this phenomenon which would be beneficial for boosting nucleic acid drug efficacy. We compared the stability of the NNA structure (ND 3) to that of soluble DNA and representative DNA–ND samples (NDs 1 and 2). For this work, we used deoxyribozymes (DNAzymes) because their catalytic activity is easily measurable, their catalytic function is fully recovered after heat inactivation, and DNAzyme activity is highly sensitive to cleavage; even hydrolysis of a single nucleotide from a DNAzyme leads to clearly detectable changes in enzyme activity.^40,44^ We exposed the NNA and DNA–ND samples to 1 U of DNase I (Figure 3e) for 2 h prior to inactivating the DNase I and assessing functional multiturnover kinetics of the DNAzyme using a dual-labeled mock RNA substrate (Table S3).^5,45^ We found that the NNA structure offered greater nuclease resistance compared to the DNA–ND samples (83% activity retained vs 66%, respectively), most likely due to steric crowding as well as the high local charge density of the nucleic acids present on the NNA.^46^ Notably, the nucleic acids used in this sequence were unmodified nucleobases. These data suggest that the ND scaffold provides enhanced protection against nucleases, further improving its potential for delivering therapeutic nucleic acids.

### NNAs are Internalized into Cells via Scavenger Receptor B1

**Figure 4:**
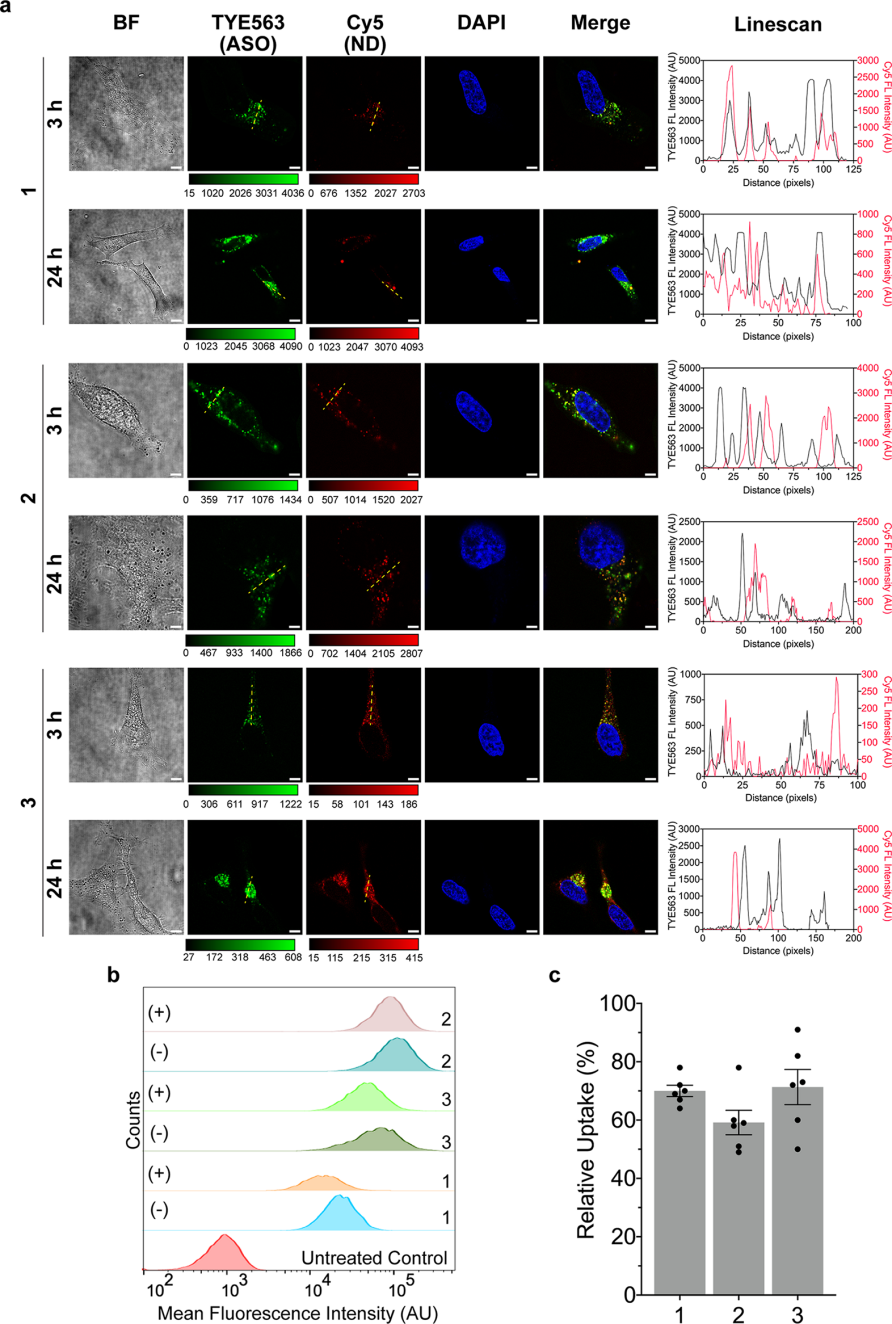
ASO–NDs and NNAs show cell uptake, which is dependent in part on scavenger receptor B1 (SRB1). (a) Representative confocal images showing a time course for the increased uptake of 100 nM ASO–NDs (NDs 1–2) and NNA (ND 3) in a time-dependent manner into HeLa cells. Panel includes brightfield (BF) image and fluorescence intensity of TYE563 (ASO) and Cy5 (1% Cy5 phospholipid for NNA). Images were taken at 3 and 24 h, and cells were fixed and stained with DAPI prior to imaging with a 60× oil objective. Scale bar: 5 μm. Linescans for the Cy5 and TYE channels are shown to the right and were generated from the yellow dashed lines shown in the images. (b) Histograms displaying the difference in uptake of representative ASO–NDs (NDs 1–2) and NNA (ND 3) samples after incubating cells with 50 μm of BLT-1 for 1 h prior to incubation with 15 nM ND (400 nM ASO) for 2 h. NDs were prepared with 0.15% Cy5 phospholipid. SRB1 uptake was compared to NDs/NNAs treated with BLT-1 (denoted as “+”) against samples treated without BLT-1 (denoted as “–”). The flow data represent mean Cy5 intensities from a minimum of 5000 cells. Raw intensity values for all the replicates are shown in Figure S3. (c) Graph comparing the uptake of the representative ASO–NDs (NDs 1–2) and NNAs (ND 3) after BLT-1 treatment. The uptake values are normalized and compared with the uptake level of samples without BLT-1 treatment as a control. This suggests that SRB1 is prominently involved in the uptake and internalization of ASO–NDs and NNAs into the cell, although it is not the only mechanism. Data represent an average of *n* = 6 independent replicates. Error bars represent SEM.

In principle, virtually any ASO can be conjugated to NDs to form NNAs, but here, we tested the clinically relevant EZN2968 HIF-1-α targeting ASO.^33^ The HIF-1-α targeting ASO was tagged with a 5′ TYE563 while the ND was labeled with 1% Cy5 to aid in quantifying cell uptake by using confocal microscopy (Figure 4a). HeLa cells were incubated with 100 nM (with respect to DNA concentration) of representative ASO–ND (1–2) and NNA (3) samples and then imaged at 3 and 24 h time points. We observed a time-dependent increase in accumulation of the NNAs and ASO–NDs as noted by the increased fluorescence signal for ND 1, 2, and 3 in confocal imaging of single cells. The lipid-Cy5 and DNA-TYE563 signals generally became more dispersed, less colocalized, and less punctate at 24 h, as shown in the images and representative linescans. These observations suggest escape of these conjugates into the cytoplasm at later time points. In contrast, at the early 3 h time point, the NNAs and ASO–NDs displayed lower total signal and more punctate clusters where the TYE563 signal was colocalized to the Cy5 signal. Moreover, the TYE563 and Cy5 signals tended to localized toward the cell edge at early time points, suggesting that a fraction of the ND is docked to the membrane^47^ or internalized and trapped inside endosomes that are near the membrane.^23^

Next, we sought to examine the mechanism of NNA uptake into the cell through flow cytometry and cell-associated fluorescence. We specifically were investigating the role of SRB1 for mediating uptake as SRB1 primarily interacts with the helices of ApoA1 and ApoA1 mimetic peptides to mediate the bidirectional transfer of cargo into the cell using a nonendocytic mechanism.^48,49^ It is plausible that ASO conjugation to the peptide would interfere with SRB1 recognition and disrupt ASO transfer, potentially diminishing the utility of the NNAs. SRB1 activity was diminished with a small molecule inhibitor named blocker of lipid transport (BLT-1).^50^ BLT-1 is well-established and binds to the key amino acid residue C384, which has a key role in selective cellular uptake of SRB1.^51^ Upon treating HeLa cells with BLT-1 (50 μM) and incubating with the panel of ASO–ND and NNA conjugates (1–5), cells were collected to assess the mean Cy5 fluorescence intensity of the ND scaffold (labeled with Cy5) via flow cytometry (Figure 4b). To our surprise, there was a significant reduction in the uptake of ASO–NDs and NNAs into cells following BLT-1 treatment for all of the NDs tested (Figure S3). This reduction correlated into 56–73% reduction in uptake for all groups (Figure 4c) when the uptake was compared and normalized against the uptake for cells treated with ASO–NDs and NNAs without BLT-1 treatment. This partial reduction in uptake of ASO–NDs and NNAs suggests that >50% of the uptake route involves other mechanisms, such as endocytosis or potentially lipid fusion with the plasma membrane. It is known that the uptake of miRNA from HDL particles is dependent on SRB1, but the mechanisms of how SRB1 mediates cholesterol and miRNA transport has not been fully elucidated.^13,14,47^ The covalent attachment of the ASO to phospholipids is not likely to limit SRB1 uptake because phospholipids are also internalized by SRB1 through the selective lipid uptake pathway during cargo transport and catabolism of the HDL scaffold.^52^ In contrast, the ND-forming peptide itself is not known to be trafficked using the SRB1 pathway, but it is likely that the amphipathic peptide–oligonucleotide conjugate may undergo internalization as noted for amphipathic cell penetrating peptides.^53,54^ While endocytosis may inevitably play a role for internalization for all nanoparticles, these experiments showing SRB1-dependent uptake enhance the therapeutic potential of NNAs as it limits endosome entrapment and eventual degradation. This data demonstrate that the C-terminal Cys-modified 22A peptide design and nucleic acid conjugation do retain the selective, nonendocytic features of the ND scaffold.

### Internalized ASO–NDs and NNAs Undergo Dissociation within 24 h

**Figure 5:**
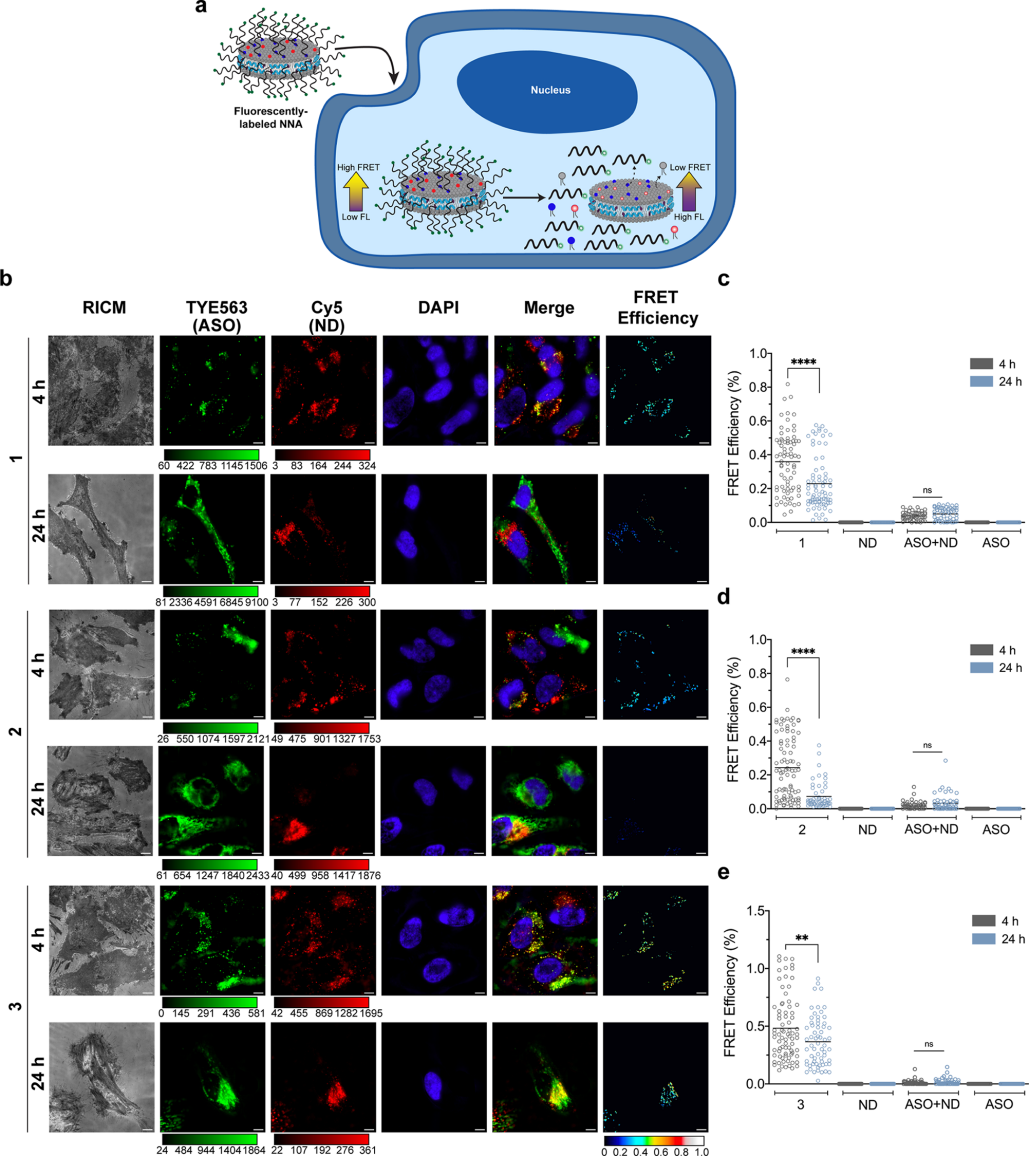
Sensitized-FRET assays reveal that ASO–NDs and NNAs measure the disassembly of the ASO from the ND scaffold within 24 h in HeLa cells. (a) Scheme detailing the mechanistic application of FRET for ASO–NDs and NNA internalization inside a cell. Conjugated ASO–NDs (NDs 1–2) and NNA (ND 3) will display low fluorescence in the donor channel because of higher FRET and vice versa is expected for ASO–NDs and NNAs once the nucleic acid detaches from the scaffold. (b) Representative epifluorescence images showing a change in FRET when cells were incubated with 100 nM ASO–NDs (NDs 1–2) and NNA (ND 3). Panel includes a RICM image and fluorescence intensity of TYE563 (ASO) and Cy5 (1% Cy5 phospholipid for NNA). Images were taken at 4 and 24 h, and cells were fixed and stained with DAPI prior to imaging with a 100× oil objective using a FRET cube. FRET images were generated from thresholding the acceptor channel (ND) and parsing the pixels and correcting for bleedthrough from the donor and acceptor (ASO). Calibration bar represents FRET efficiency (set between 0 and 1.0) Scale bar: 5 μm. Plot of FRET efficiencies for representative samples of the ND scaffold for DNA–ND 1 (c), DNA–ND 2 (d), and NNA 3 (e) depicting a decrease in FRET over time from 4 to 24 h. The NNA sample (ND 3) demonstrated a higher FRET efficiency at 24 h compared to ASO–NDs (1–2), suggesting that it takes a longer time for the ASO to dissociate from the ND scaffold. ND mixed with ASO, ASO only, and ND only were used as controls and showed no FRET efficiency as they were not conjugated to a scaffold. Epifluorescence microscopy images are shown in Figure S4. An average of *n* = 30 cells were analyzed for each sample; ** *p* < 0.01, **** *p* < 0.0001.

To better characterize the integrity of ASO–NDs and NNAs after internalization into the cell, we used sensitized-FRET^55^ to determine lipid-nucleic acid proximity (Figure 5a). Similar to the uptake studies, HeLa cells were incubated with 100 nM ASO–NDs (1–2) and NNA (3) for 4 and 24 h. In this experiment, similar to our previous FRET experiment, we expected that we would observe a high FRET efficiency (due to nanoscale proximity of DNA to ND and peptides) at 4 h compared with 24 h, where some disassembly is expected for the conjugated NNAs and DNA–NDs. After rinsing and nuclear staining of the cells, the samples were imaged on an epifluorescence microscope using a FRET cube equipped to measure FRET using the TYE563 and Cy5 wavelengths (Figure 5b). The dyes used for these sets of experiments were specifically chosen because their quantum yields are fairly pH insensitive. FRET images were acquired at an exposure time of 200 ms to avoid photobleaching. FRET efficiency was determined by using a pixel-by-pixel analysis method (Figure S4, eqs 5–9) that accounted for cross talk between the donor and acceptor channels.^56^ For all the ND sample types that were examined, we noticed a decrease in FRET efficiency when we compare values at 4 and 24 h (Figure 5c–e). This confirms that ASO–ND conjugates dissociate over this time window. As expected, there was no detectable FRET for the ND scaffold only, DNA only, and the ND mixed with the DNA at the 4 and 24 h time points (Figure S5). It is important to note that because the donor and acceptor are not directly linked to the same molecule, the FRET efficiency for our ND conjugates is inherently lower due to a larger Förster radii and especially for the ASO–ND 2 where the ASO is conjugated to the scaffolded peptide. The FRET efficiencies measured for all samples at *t* = 4 h were 36% for ASO–ND 1, 24% for ASO–ND 2, and 48% for NNA 3. There was not sufficient uptake of ND at *t* = 0 to quantify DNA–ND and NNA integrity at early time points. Thus, we next measured FRET efficiency at 24 h and found values of 23% for ASO–ND 1, 7% for ASO–ND 2, and 37% for NNA 3. This data show that the ASO–ND and NNA assemblies are gradually dissociating. The disassembly of ASO–ND constructs is due to the activity of a combination of proteases, lipases, and nucleases as well as the retro-Michael (maleimide–thiol) reaction which releases the ASO from the ND and occurs under physiological conditions in the cytoplasm (i.e., highly reducing environment from glutathione).^57^ Note however that for activity purposes it is not required that the ASO be strictly localized to the NNA and, in fact, release from the scaffold can enhance the activity of ASO in the cell to bind mRNA, recruit RNaseH, and block the ribosome.

### Quantifying NNA and ASO–ND Activity *in Vitro*

**Figure 6:**
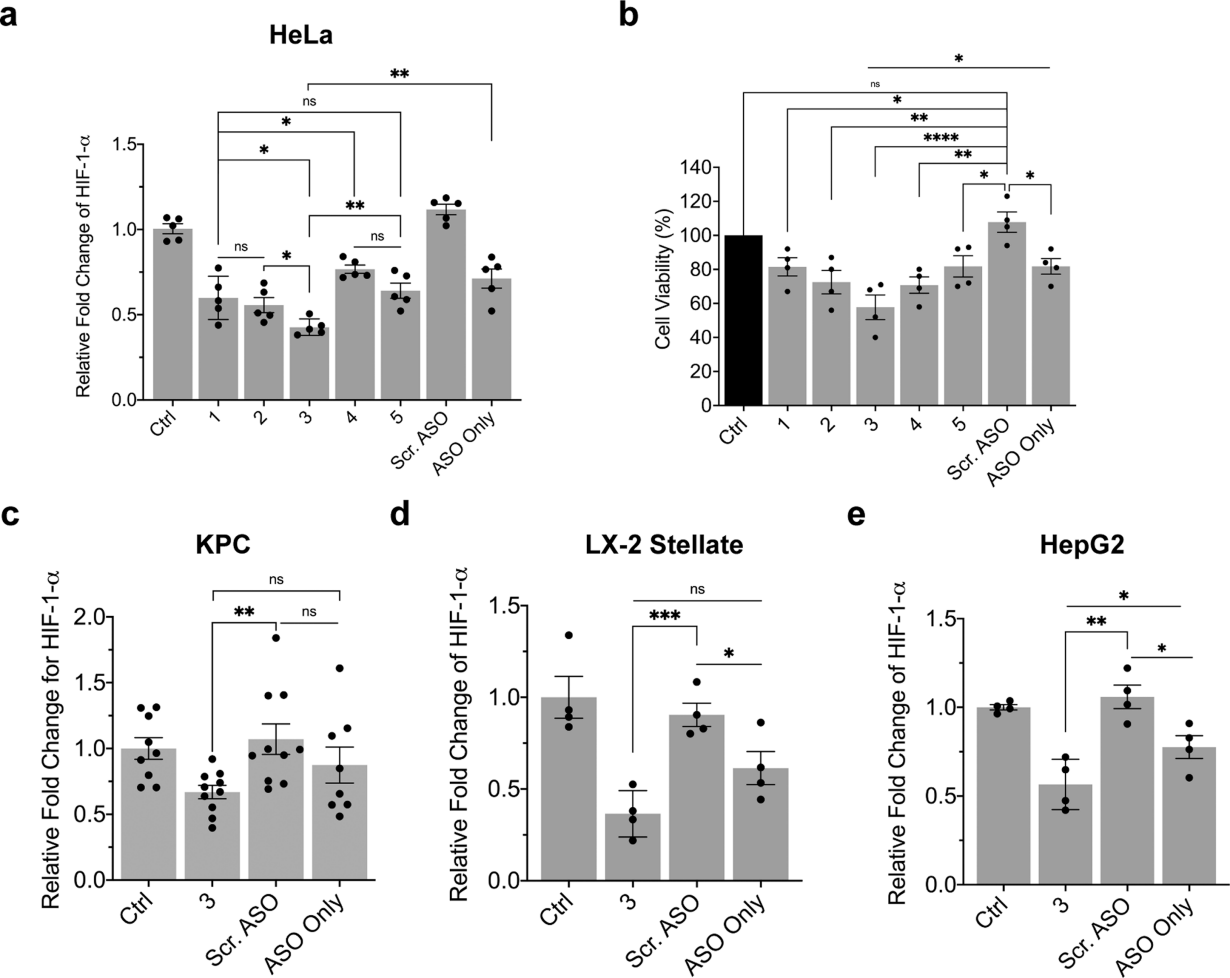
Quantifying the functional activity of ASO–NDs and NNAs for reducing HIF-1-α mRNA levels in different cell lines. (a) Plot of HIF-1-α levels after incubating HeLa cells with ASO–NDs (NDs 1–2, 4) and NNAs (NDs 3 and 5) for 24 h prior to extracting mRNA and quantifying the levels via RT-qPCR. The anti-HIF-1-α ASO (EZN2968) was used for this study, and the ASO concentration was 100 nM for all groups, including the scrambled (scr.) ASO (conjugated on ND) and ASO only. The ASO–ND and NNA conjugated to the peptide on the C-terminus (NDs 2–3) had greater activity compared to the N-terminus (NDs 4–5). Data represents an average of *n* = 5 independent replicates. (b) Assessment of cell viability using the MTT assay. HeLa cells were subject to 100 nM ASO–ND or NNA and incubated for 24 h prior to adding MTT reagent and performing the assay. The values are normalized to the OD measured at 590 nm for the untreated cells as a control (ctrl). Cells treated with ASO–ND or NNAs demonstrated a significant reduction in viability, further confirming the functional activity of the conjugates. Each data point represents the percent viability for one replicate (*n* = 4 independent replicates). Quantification of HIF-1-α levels as determined via RT-qPCR in (c) KPC, (d) LX-2 Human Hepatic Stellate, and (e) HepG2 cells after incubating cells with 100 nM NNA (ND 3), scrambled (scr.) ASO on a ND, and ASO only for 24 h. Transcript levels are normalized to the untreated control group (ctrl). The NNA scaffold significantly boosted ASO activity compared to cells administered 100 nM ASO only without a scaffold. Data represent an average of a minimum of *n* = 4 independent replicates; * *p* < 0.05, ** *p* < 0.01, *** *p* < 0.001, **** *p* < 0.0001. Error bars represent SEM.

In these sets of experiments, we aimed to compare the activity of the ASO conjugated NDs and NNA scaffolds by measuring HIF-1-α transcript levels using qPCR.^58^ HeLa cells were incubated with 100 nM ASO–ND or NNA for 24 h before cells were lysed and RNA was collected for qPCR. All treatment groups had an ASO concentration of 100 nM, and this was validated using the extinction of DNA at λ = 260 nm. In general, we noted that the NNA scaffolds showed activity toward reducing basal HIF-1-α mRNA levels (Figure 6a). Of the constructs tested, ND 2 and ND 3 (NNA) showed greater levels of activity compared to ND 4 and ND 5 (NNA). In other words, conjugation of the ASO to the C-terminal cysteine of the ApoA1 mimetic peptide generated NDs that were significantly more active than ASO’s conjugated to the N-terminus of the peptide. Overall, NNA (ND 3) showed the greatest level of activity (58% reduction of cellular HIF-1-α levels), suggesting that creating a high density of ASO around the ND scaffold leads to improved activity per ASO. ASO–ND 4 showed the least activity (24% knockdown), and the relatively lower activity of ND 4 and ND 5 (NNA) is attributed to the destabilizing effects (and larger aggregates) of linking the DNA to an N-terminal Cys that resides in the hydrophobic face of the peptide, as suggested by the helical wheel diagram (Figure S1). This lower activity is further supported by TEM analysis and DLS that showed more broadly distributed NDs and a subpopulation of lipid assemblies with >100 nm (Figure S2). Because cancer cells typically have an overreliance on glycolysis (Warburg effect) and HIF-1-α expression maintains upregulated glycolysis levels, knockdown of HIF-1-α can contribute to reducing cell viability.^59^ We thus validated the qPCR quantification of HIF-1-α by also measuring cell viability of HeLa cells treated with 100 nM ASO–NDs and NNAs for 24 h using an MTT assay (Figure 6b). Overall, all ASO–ND and NNA conjugates used for this study displayed significant reduction in cell viability when compared against treatment with a scrambled ASO. Consistent with the qPCR results, NNA (ND 3) showed the greatest reduction in HeLa cell viability (42% reduction) compared to the other scaffolds (1–2, 4–5). Taken together, this data identifies NNAs using ND 3 as the most potent scaffold for gene regulation.

To validate the activity of NNAs in other cell types, we next tested HIF-1-α knockdown in three other model cell lines, including KPC (Figure 6c), LX-2 human stellate (Figure 6d), and HepG2 (Figure 6e). These cell lines were chosen because of their intrinsic overexpression of HIF-1-α and represent different disease models (e.g., KPC: pancreatic ductal adenocarcinoma; LX-2: hepatic fibrogenesis in NAFLD; HepG2: hepatocellular carcinoma) which are often exacerbated by abnormal levels of hypoxia. In each of these cell lines, there was a significant decrease in cellular HIF-1-α levels when treated with 100 nM NNA (ND 3) for 24 h compared to the scrambled ASO. The NNA treatment displayed slightly more activity compared to that of soluble ASO only. This can be explained by the fact that phosphorothioate-modified ASOs are usually trafficked into cells using multiple nonproductive endocytic pathways depending on the cell surface protein present for internalization, hence suggesting that functional ASO uptake is enhanced when placed on a ND scaffold.^60^ These data conclude that the NNA conjugate prepared from peptide B is active *in vitro*, as we can confirm from validating its activity in multiple cell lines.

### NNAs Penetrate the Hypoxic Core of Tumor Spheroids and Display Activity

**Figure 7:**
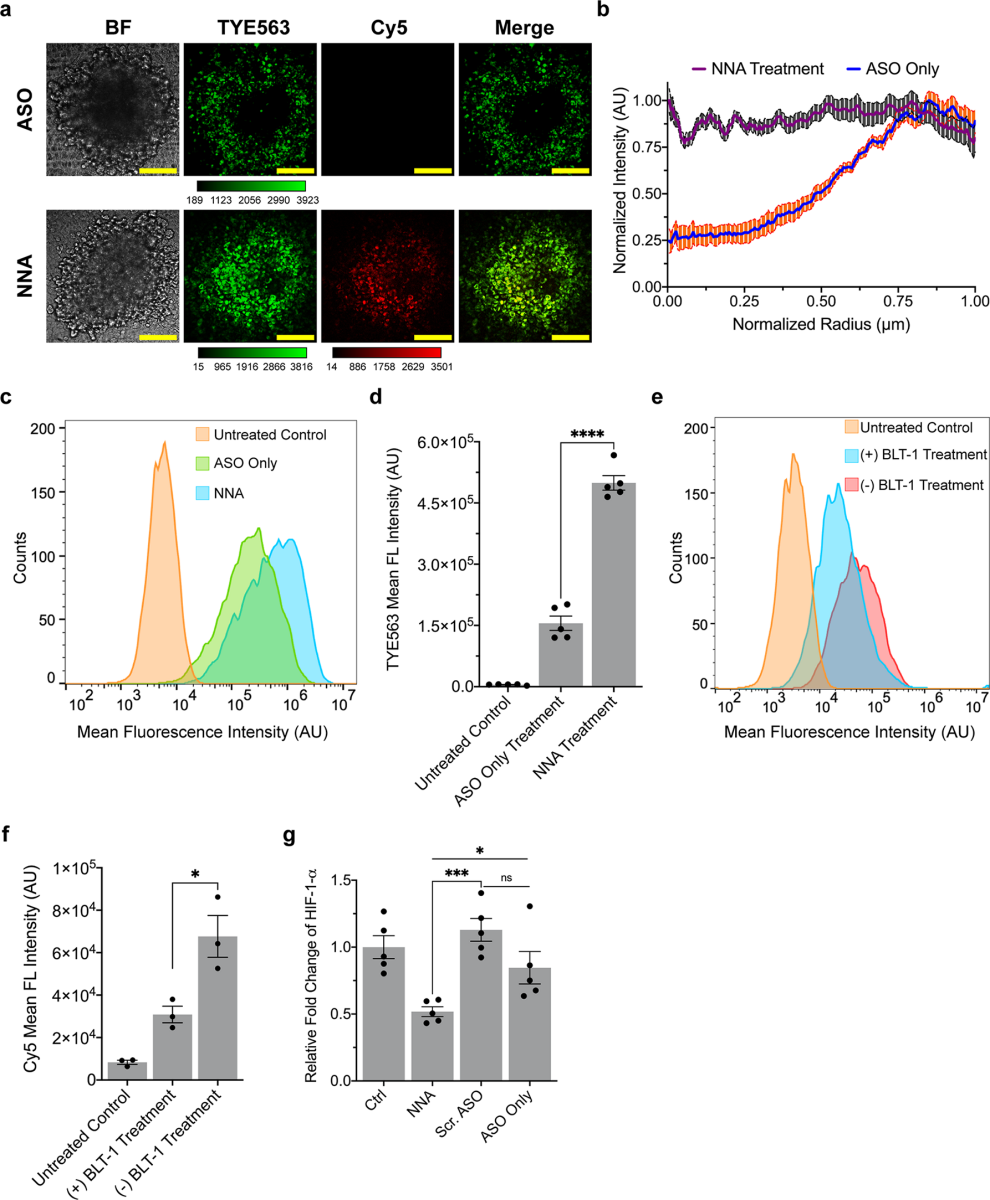
NNA uptake and functional activity in H1299 3D spheroids. (a) Confocal images of internalized NNAs and ASO (EZN2968) visualized inside the spheroid cross section at the middle slice after 24 h of incubation (ASO concentration = 100 nM). Panel includes bright-field (BF) image and fluorescence intensity of TYE563 (ASO) and Cy5 (NNA). Scale bar: 15 μm. (b) Radial profile scan averaging TYE563 fluorescence intensity and normalizing for 14 spheroids (*n* = 3 independent replicates). The profile scan reveals that the NNA uptake is more enhanced for spheroids with the ability to penetrate the hypoxic core within 24 h. (c) Representative flow cytometry histogram depicting cell uptake when comparing between the spheroids treated with ASO only and the NNA (ND 3) treatment. The flow data represent mean TYE563 intensities from a minimum of 5500 cells. (d) Mean TYE563 fluorescence quantification on the uptake of ASO and NNA (ND 3) as measured via flow cytometry. Each data point represents the average of 6 spheroids (*n* = 3 independent replicates). (e) Representative flow cytometry histogram depicting the difference in the uptake of NNAs into spheroids after blocking SRB1 with 50 μm of BLT-1 for 1 h. NNAs (150 nM ASO and 6 nM ND) were subsequently incubated with spheroids for 2 h prior to dissociating the spheroids into individual cells and measuring cell associated fluorescence using flow cytometry. The flow data represents mean Cy5 intensities from a minimum of 5000 cells. (f) Mean Cy5 fluorescence quantification for the uptake of NNA (3) into spheroids treated with BLT-1. There is a 54% reduction in the uptake of NNAs after the BLT-1 treatment. Each data point represents an average of 8 spheroids (*n* = 3 independent replicates). (g) Plot comparing the HIF-1-α levels as measured by RT-qPCR after subjecting the spheroids to 550 nM ASO, ASO on NNA, or scrambled (scr.) ASO on a ND for 24 h. Transcript levels are normalized to the untreated control group (ctrl). Each data point represents an average 5 spheroids (*n* = 3 replicates); * *p* < 0.05, ** *p* < 0.01, *** *p* < 0.001, **** *p* < 0.0001. Error bars represent SEM.

As a stepping stone toward *in vivo* validation of NNA activity, we next employed a cancer spheroid model as a representative tumor model. Tumors typically consist of a poorly oxygenated and poorly vascularized necrotic core.^61^ The highly hypoxic core presents with diffusional selectivity, and most drugs face mass transport barriers which limits delivery to the necrotic core.^62^ One would expect that larger scaffolds and higher molecular weight drugs, such as NNAs, would experience significant barriers to reaching the core of tumor spheroids. On the other hand, tumors and other malignant cell lines highly express SRB1, thus making it possible to deliver cargo into the spheroid. To test whether NNA can penetrate the hypoxic core of spheroids, we used the most active NNA (ND 3) in subsequent experiments with the three-dimensional H1299 nonsmall cell lung cancer (NSCLC) spheroid model. Spheroids embedded in Matrigel were incubated with 100 nM NNA or ASO for 24 h before visualizing using confocal microscopy (Figure 7a). Representative images show that there was a marked increase in NNA uptake into the core of the spheroid compared with the ASO only treatment (Figure S6). The soluble ASOs tended to internalize into the quiescent and proliferating areas (closer to surface and edges) of the spheroid. This was quantified by running a radial profile analysis of *n* = 14 spheroids (Figure 7b). The radial scans were initiated from the center of the spheroid out to the edge as determined from the brightfield image. The radial profile of soluble ASOs showed a weak signal in the core of the spheroid and a gradual increase before leveling off in the proliferating zone. Conversely, NNA treatment shows a rather uniform level of ASOs throughout the spheroid, highlighting the deeper penetrative ability of these discs. This confocal analysis strongly suggests that NNAs would provide a more effective strategy to deliver larger doses of ASOs to tumors. To quantify total uptake, we next used flow cytometry to compare ASO uptake when the ASO was linked to the NNA compared to soluble DNA. We found that NNA uptake was significantly greater than that of the ASO only treatment when directly evaluated using flow cytometry (Figure 7c,d). Thus, NNAs offer a significant improvement in delivery to tumor spheroids in terms of both total uptake and delivery to the hypoxic core.

We next aimed to test the role of SRB1 in mediating the penetration of NNA to the necrotic core of spheroids. Here, we treated spheroids with 50 μM BLT-1 for 1 h before adding 6 nM Cy5-ND (150 nM ASO) for 2 h. Spheroids were dissociated into individual cells, and the Cy5 fluorescence was measured using flow cytometry (Figure 7e). There was a 54% (*p* < 0.0001) decrease in uptake in the spheroids treated with BLT-1 compared to untreated spheroids (Figure 7f). This confirms that SRB1 plays a major role in NNA mediating delivery of ASOs to spheroids. This was not unsurprising, as other work suggests an important role for SRB1 in ND uptake.^63^ Furthermore, the small size of the ND, compared to other types of nanoparticles such as liposomes, greatly expediates transport across the extracellular matrix and interstitial openings.^64^

Lastly, we tested the activity of anti-HIF-1-α NNA by treating the spheroids with 550 nM ASO or NNA for 24 h. Treatment of spheroids at this dosage resulted in an average 49% reduction in cellular HIF-1-α levels (Figure 7g). Additionally, spheroids treated with just ASO resulted in only a 16% reduction in cellular HIF-1-α mRNA. The NNA conjugate exhibited significant potency against the ASO only treatment, hence signifying the potential for using NNAs to deliver nucleic acids as a form of cancer therapy for mediating hypoxia and sensitizing malignant tumors for an increased response from other drug treatments.

### Anti-HIF-1-α NNAs Are Active *in Vivo*

**Figure 8:**
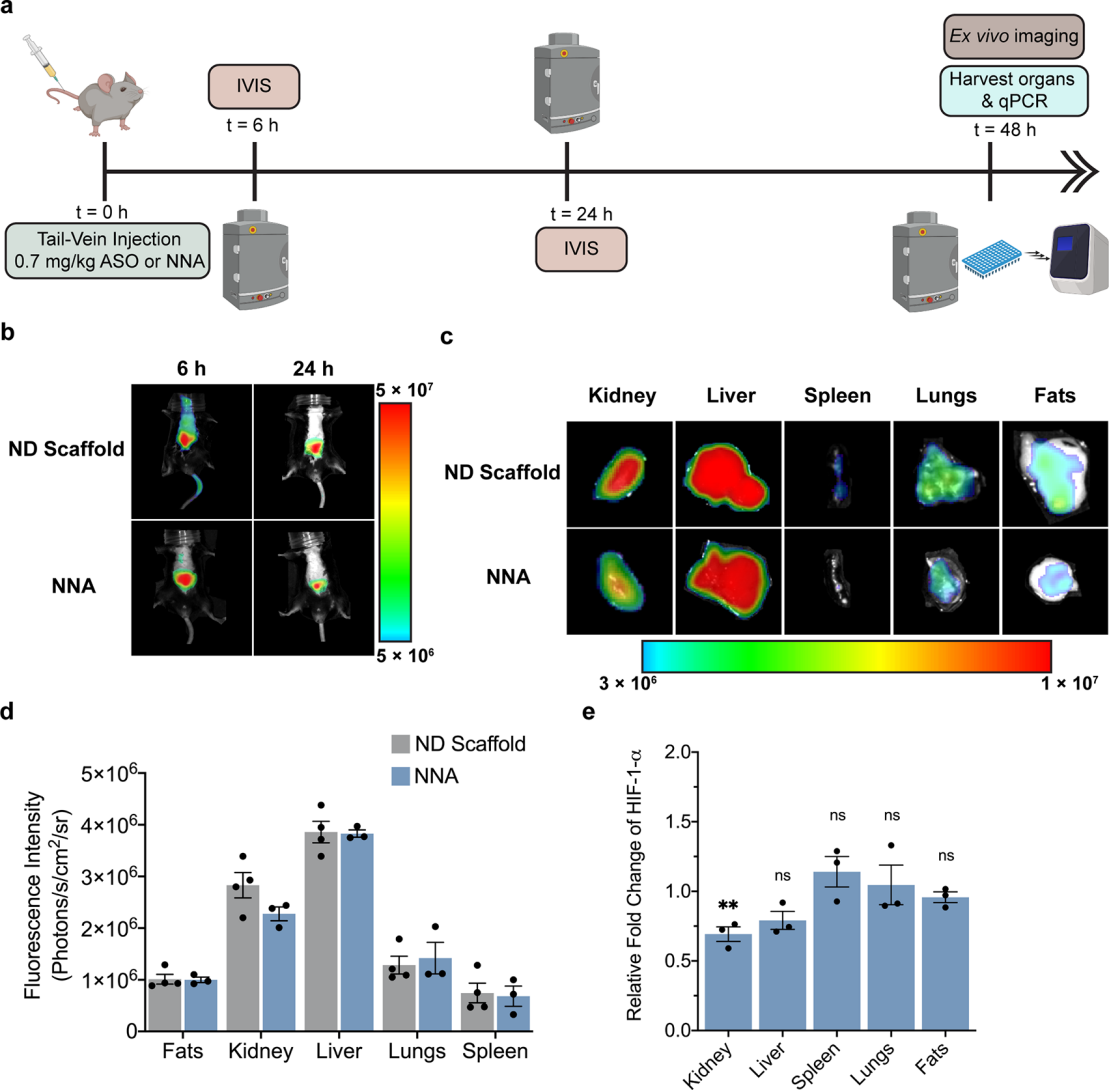
*In vivo* internalization and gene-silence efficacy of NNAs. (a) Timeline of *in vivo* imaging and qPCR experiments after injecting C57BL/6 mice with NNA, ND scaffold, and ASO only samples. Mice are injected with 0.7 mg/kg HIF-1-α ASO drug and sacrificed for *ex vivo* imaging and organ harvesting for RT-qPCR. (b) *In vivo* fluorescence imaging for the uptake of the ND scaffold and NNA (ND 3) in C57BL/6 mice (*n* = 4). Representative images of mice were taken at 6 and 24 h postinjection. (c) *Ex vivo* fluorescence imaging of mice injected with an ND scaffold (ND 3) and NNA (ND 3). Organ imaging was performed at 48 h postinjection on the kidneys, liver, spleen, lungs, and fats. (d) Plot quantifying the fluorescence intensity measured for the different organs of the mice from the *ex vivo* imaging. Increased uptake is correlated with increased fluorescence, and this was primarily observed in the kidneys and liver, followed by some observable uptake in the lungs. (e) Quantification of HIF-1-α levels in the harvested organs via qPCR after treatment with NNA (ND 3) for 48 h. The data was normalized against identical tissues treated with the ND. Error bars represent SEM. Each data point represents the result from one single animal normalized to animals treated with ND scaffold; ** *p* < 0.01.

We next aimed to test whether NNAs are active *in vivo* using a mouse model. We designed the experiment, as shown in Figure 8a, where a single tail-vein injection of the NNA or ND scaffold was subsequently followed 48 h later with analysis of HIF-1-α gene expression in different tissues. EZN2968 anti-HIF-1-α ASO was conjugated to the NNA as describe above, purified, and then quantified by UV–VIS to determine the concentration. We delivered 200 μL of approximately 1 μM NNA and ND solutions that were doped with 1% Cy5 phospholipid. The DNA concentration was approximately 13 μM which is equivalent to a dose of 0.7 mg/kg ASO into C57BL/6 mice by tail-vein injection (Figure 8a). Live trafficking of the NNA and ND scaffold was visualized *in vivo* using a whole-body imaging at *t* = 6 and 24 h postinjection (Figure 8b). Most of the localization of the NNA at *t* = 6 h was in the abdominal area (liver and kidneys), with some minor accumulation near the bronchial area. In contrast, the ND control scaffold was more uniformly distributed and was still present in the tail-vein at the 6 h time point. This more rapid localization of NNAs to the liver/kidney was maybe due to the result of the ASO, which increases the molecular weight and hydrodynamic size of the particles, which also increases cell uptake.^65−67^ Within 24 h, the injected ND scaffold and NNA were localized primarily to the abdominal area. At 48 h postinjection, organs were harvested for *ex vivo* imaging (Figure 8c). The NNA conjugates accumulated in liver, kidney, spleen, lung, and fat tissues, which was also noted for the ND. The amount of uptake as inferred from the total fluorescence intensity of the tissues indicated comparable levels for the ND and NNA. Fluorescence quantification detailed that the kidney and liver were the two major organs for internalizing the NNA and ND scaffold (Figure 8d). This is consistent with prior research reporting ND uptake.^63,68^ RNA was extracted from harvested organs, and the relative HIF-1-α levels in each organ were evaluated through qPCR. We noted knockdown of HIF-1-α following NNA treatment in the liver and kidney tissues, with the kidney knockdown showing statistical significance (*p* < 0.01). Importantly, tissues that showed weaker uptake, such as the spleen, lung, and fat, showed no observable decrease of HIF-1-α as was expected (Figures 8e and S7). These results are similar to experiments reported by Enzon Pharmaceuticals^33^ where a 3.6 mg/kg dose injected daily over a 2-week time frame was the minimum that resulted in significant knockdown of HIF-1-α in kidney and liver tissues. However, dosing at 0.7 mg/kg with daily injections of soluble ASO did not show significant knockdown in the Enzon experiments, suggesting that the NNA conjugation increases efficacy of the therapeutic (∼5-fold lower than the minimum effective dose used in the parent study) over a shorter time frame (2 days vs 14 days).^69^ This data further confirms the therapeutic potential of NNAs. Aside from lowering the effective dose required (and, by extension, reducing patient side effects that can result from oligonucleotide drugs), the direct targeting features bestowed by the NNA may reduce the overall time required for treatment.

## Conclusion

In this study, we developed strategies to maximize the loading of DNA onto ND scaffolds. The innovation in this work pertains to two concepts. The first is the idea of conducting a one-pot reaction to covalently conjugate DNA to the peptides and phospholipids comprising the ND assembly by using an efficient Michael addition. Second, we recognized the pitfalls associated with introduction of a thiol into the HDL mimetic peptide 22A and thus screened for Cys sites that minimally perturb the amphipathic character of the peptide and its capacity to self-assemble into ND in the presence of phospholipids. The optimal resulting NNA construct was assembled with one cysteine insertion on the C-terminus of the peptide, 90% DMPC phospholipid, and 10% Ptd-thioethanol. DNA conjugation afforded an average of 30 copies of DNA covalently bound per NNA and still retained sub-30 nm diameters and morphologies identical to those of the parent ND scaffold. NNAs demonstrated a high degree of nuclease protection, including DNase I. The NNA assembly did not alter the selective targeting properties of the scaffold, as uptake was discovered to be partially SRB1 dependent. Furthermore, we learned through sensitized-FRET that the ASO does separate from the scaffold within 24 h of incubation and uptake within cells. The NNA containing ASO modified to the C-terminus was identified as the most active *in vitro* as determined by qPCR and viability studies using HeLa, KPC, Lx-2 Stellate, and HepG2 cells. The NNA construct, owing in part to its smaller size and its specific targeting features, showed penetration into the necrotic core of a 3D H1299 cancer spheroid model which was correlated with greater levels of antisense activity compared to that of soluble ASOs. The NNAs are highly potent *in vivo* where we visualized and quantified higher uptake in the liver and kidneys, correlating to the higher activity for reducing HIF-1-α mRNA levels using only a single low dose (0.7 mg/kg) of the ASO drug. Adopting the NNA platform may offer a compelling solution to bolstering the oligonucleotide therapeutic efficacy in the clinic. Linking a single-stranded oligonucleotide can also enable us to build structured material assemblies of nucleic acid coated NDs, which can provide optimal dosing and further improve the therapeutic response associated with nucleic acid drugs. Overall, the versatile and advantageous platform presented by the NNA can enable us to precisely tune our scaffold to help significantly expand delivery efficiency and improve therapeutic outcomes for multiple drug targets across the board.

## Materials and Methods

Lipids used to prepare ND scaffolds were purchased (Table S2, Figure 1c) from Avanti Polar Lipids (Alabaster, AL, USA), including DMPC, Ptd-thioethanol, and Cy5-PE. Oligonucleotides, including the DNAzyme and ASO, and HIF-1-α and 18S primers for RT-qPCR were custom-synthesized (Table S3) by Integrated DNA Technologies (Coralville, IA, USA). The modified and unmodified 22A ApoA1 mimetic peptides (Figure 1b) were purchased from Genscript (Piscataway, NJ, USA) with N- and C- termini capped with acetylation and amidation, respectively, and TFA salts were removed. TEM samples were prepared on 400 mesh carbon-coated copper grids obtained from Electron Microscopy Sciences (CF400-Cu; Hatfield, PA, USA), and negative staining was performed with Nanoprobe’s Nano-W (Yaphank, NY, USA). SMCC (22360), DNase I (EN0521), 6× Loading dye (R0611), and Bond-Breaker TCEP Solution (77720) were obtained from ThermoFisher Scientific (Waltham, MA, USA). Organic solvents used for SMCC coupling including anhydrous *N*,*N*-dimethylformamide (DMF, 227056) and *N*,*N*-diisopropylethylamine (DIPEA, 496219) were purchased from Sigma-Aldrich (St. Louis, MO, USA). DNA stock solutions, buffers, and other aqueous experiments were prepared by using water from a Barnstead Nanopure Water System from ThermoFisher Scientific (Waltham, MA, USA) at a resistivity of 18.2 MΩ. Ethanol precipitation to separate unreacted SMCC from DNA utilized Koptec’s USP-grade 200-proof ethanol from Decon Laboratories (V1001; King of Prussia, PA, USA) and sodium acetate (3 M, pH 5.2, molecular biology grade) from MilliporeSigma (567422; Burlington, MA, USA), and a hydrated P-2 gel from Bio-Rad (1504118; Hercules, CA, USA) was used for purification. Unreacted maleimide DNA from NNA, ASO–ND, and DNA–ND conjugation was removed using size exclusion with a MWCO: 50 kDa Amicon Filter (UFC505024), Blocker of Lipid Transport-1 (SML0059), and Omnipur Agarose (2120-OP) purchased from EMD Millipore (Burlington, MA, USA). Quant-iT OliGreen ssDNA Reagent (O7582) and nuclear DAPI stains NucBlue Fixed Cell ReadyProbes (R37606) and SYBR Gold Nucleic Acid Gel Stain (S11494) were purchased from Invitrogen (Carlsbad, CA, USA). Cells were cultured in Dulbecco’s Modification of Eagle’s Medium with l-Glutamine (DMEM, 10-013-CM) and supplemented with Fetal Bovine Serum (FBS, 35-010-CV) and Penicillin–Streptomycin (30-002-CI) and were detached using Trypsin EDTA (25-053-Cl) from Corning (Tewksbury, MA, USA). H1299 cells were cultured in RPMI-40 with l-Glutamine (11875093) acquired from Gibco (Waltham, MA, USA). Spheroids were initially seeded in ultralow attachment microplates (07-201-680) and later implanted in Matrigel Basement Membrane (356255 and 354234), and both items were purchased from Corning (Tewksbury, MA, USA). Spheroids for microscopy imaging were embedded in Matrigel on 35 mm Mattek plates with a No. 1.5 glass coverslip with a 14 mm diameter (P35G-1.5-14-C) purchased from Mattek corporation (Ashland, MA, USA). qPCR was performed with the following: RNeasy Mini Kit (74106) and QIAzol (79306) from Qiagen (Hilden, NRW, Germany), High-Capacity cDNA Reverse Transcription Kit from Applied Biosystems (4368814; Foster City, CA, USA), and PerfeCTa SYBR Green FastMix Reaction Mix from QuantaBio (101414-278 [VWR]; Beverly, MA, USA). C57BL/6 mice were purchased from Jackson Laboratories (Bar Harbor, ME, USA). Prior to *in vivo* imaging, hair was removed using Nair (Ewing Township, NJ, USA) and mice were anesthetized using isoflurane (DP7000) from Dechra Pharmaceuticals (Northwich, Cheshire, United Kingdom).

### Synthesis and Characterization of NDs and NNAs

DMPC and thiol phospholipid stocks in chloroform were further diluted with chloroform in a 90:10 molar ratio. The mixture was evaporated using rotary evaporation and allowed to dry for 30 min. For fluorescent experiments, Cy5-PE was doped in at a molar ratio of 0.15% or 1% as necessary. After 30 min, the lipid mixture was placed under a steady stream of nitrogen for 10 min, and the lipid film was hydrated with phosphate buffered saline (PBS, pH 7.4). The hydrated mixture was sonicated for 10 min and subjected to 3 freeze–thaw cycles to ensure all the lipids were incorporated. SUVs were then prepared by passing the mixture 5 times through a 10 mL LIPEX thermobarrel extruder (Evonik Industries, Essen, Germany) using an 80 nm polycarbonate filter. ApoA1 mimetic peptides (A, B, C, or D) were dissolved (2 mg) in nanopure water and combined with the SUVs. To assist in solubilization, the peptide–SUV mixture was vortexed for 15 s prior to performing three warm–cool cycles alternating between 55 and 4 °C for 15 min. The ND scaffolds (1–7) were stored for a maximum of 3 weeks at 4 °C.

### Size and Morphology Characterization of NDs and NNAs

NDs and NNAs were measured as is (no further dilution) by using DLS on a NanoPlus DLS Nano Particle Size and Zeta Potential Analyzer (Micromeritics Instrument Corporation, Norcross, GA, USA) instrument. Sample preparation for TEM was prepared as previously described.^23^ Briefly, 400-mesh copper grids were plasmon-etched, and a 5 μL drop of sample was placed on the grid for 30 s before gently blotting with a KimWipe and allowing the sample to dry for 2 min. One drop of Nano-W was applied to the dried grid for another 2 min before blotting and drying. Samples were visualized using TEM on a Hitachi HT7700 instrument (Chiyoda City, Tokyo, Japan) operating at 80 kV accelerating voltage. Some images were collected using a JEOL-JEM TEM microscope (Peabody, MA, USA) operating at 120 kV accelerating voltage when available. TEM images were analyzed by using Fiji.

### Coupling Maleimide-DNA onto the ND and NNA Scaffolds

Maleimide-DNA was prepared as previously described.^23^ Briefly, SMCC (2 mg) was dissolved in DMF and DIPEA (0.2-fold v/v) and combined with amine-modified DNA (1 mM) for 1 h at RT. Unreacted SMCC was removed through ethanol precipitation and a P2 gel in a size exclusion column. The maleimide-DNA was purified via HPLC on an AdvanceBio Oligonucleotide (Santa Clara, CA, USA) column to remove any unreacted DNA. Samples were dried in a vacuum centrifuge overnight before coupling. Coupling was performed by reducing any disulfide linkages present in the peptide and/or lipid using 10-fold excess TCEP. After removing TCEP with an Amicon (MWCO: 50 kDa) column and calculating the ND concentration as described previously,^23^ purified SMCC-DNA was dissolved in PBS (pH 8.5) and combined in 12-fold excess with the ND and NNA scaffolds. The reaction was performed by gently agitating the mixture at 45 °C for 2 h before removing unbound DNA using centrifugal filtration with an Amicon size exclusion column (MWCO: 50 kDa) at 8500 rpm for 7 min and washing 4 times (or until the supernatant displayed no 260 nm absorbance) in between with fresh PBS (pH 7.4) After the final wash, the solution was recovered (recovery volume 30–50 μL) and stored at 4 °C until further use.

### Quantifying DNA Density

DNA density was quantified using the commercially available OliGreen kit as described previously.^23^ In summary, a stock solution of DNA (12 μg/mL) that contained the same sequence strand as the sample was used to prepare a calibration curve at the following concentrations: 0.01, 0.1, 0.2, 0.5, 0.75, and 1 μg/mL in 1× TE buffer. The diluted DNA calibration samples were supplemented with NDs and trypsin to facilitate disassembly. ND and NNA samples were prepared at the following concentrations: 0.2, 0.4, 0.6, and 1.2 nM in 1× TE buffer with trypsin. Samples were heated to 85 °C for 10 min before adding OliGreen and measuring the fluorescence intensity (Ex/Em = 485/528 nm) on a Synergy H1 Biotek Plate Reader (Winooski, VT, USA). DNA density values were calculated by dividing the total concentration of DNA on the DNA–ND/NNA measured by 260 nm absorbance and Beer’s Law or as determined through a standard curve generated from the OliGreen assay and then divided by the ND concentration (eqs 1–3). For equation 1, the radius, *r*, can be directly obtained from the ND diameter measured from TEM. The DMPC lipid footprint value, *p*, used for calculation purposes was 0.63 nm^2^.
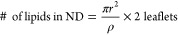
1
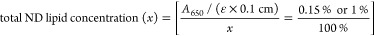
2
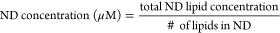
3

### Bulk Solution FRET Measurements of DNA–NDs and NNAs

DNA (HIF-1-α ASO, Table S3) was fluorescently labeled with TYE563 and then conjugated to the ND scaffolds containing 1% Cy5 phospholipids. DNA–ND and NNA samples were prepared by diluting the stock solution to a final concentration containing 100 nM DNA. As controls, 100 nM DNA mixed with ND (but unconjugated) and 100 nM DNA only were prepared in PBS. The donor fluorescence was measured on a fluorometer from Horiba Scientific (Edison, NJ, USA) by exciting the samples at λ = 525 nm, and emission spectra were collected with 20 accumulations and a 0.1 integration measured at λ = 563 nm. FRET efficiency was calculated as shown in eq 4.
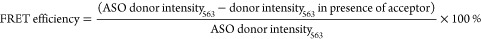
4

### Gel Electrophoresis and Serum Degradation Assay

A 1.5% agarose gel was prepared using in-gel staining with SYBR Gold (10 000× dilution). ND scaffolds were prepared with 0.15% Cy5 phospholipid to enable detection during the fluorescence readout. Samples (5 μL) containing unbound DNA (control), DNA–NDs and NNAs (1–5), and unconjugated DNA mixed with ND were mixed with 6× loading dye (5 μL), and samples (10 μL) were loaded onto the gel; the gel was run at 85 V for 1.5 h on a Bio-Rad PowerPac Basic Electrophoresis Supply (Hercules, CA, USA) and visualized using an Amersham Typhoon laser scanner (Cytiva, Marlborough, MA, USA).

### DNase I Assay

NDs and NNAs containing DNAzyme along with soluble DNAzyme were diluted to 1 μM (DNA concentration) using HEPES buffer (10 mM HEPES, 150 mM NaCl, pH 7.4) and combined with 1 U of DNase I (or an additional buffer for the control samples). The samples were incubated at 37 °C for 2 h. Thereafter, DNase I was quenched using the supplied EDTA (5 mM) and inactivated at 65 °C for 10 min, as per the accompanied protocol. The samples were transferred to a 96-well plate and combined with the DNAzyme substrate solution mix (10 mM HEPES, 150 mM NaCl, 10 μM DNAzyme substrate, 4 mM MgCl_2_, pH = 7.4), and FAM fluorescence intensity was immediately monitored for 2.5 h using the plate reader. DNase I degradation was determined by comparing the fluorescence intensity at 2.5 h of the DNase I treated samples against the same sample that did not contain DNase I.

### Cell and Spheroid Culture

HeLa cells (ATCC), HepG2 cells (ATCC), and KPC cells were cultured in DMEM containing l-glutamine with 10% FBS and 1% penicillin (100 U/mL) and streptomycin (100 mg mL^–1^). LX-2 Human Hepatic Stellate cells (Sigma-Aldrich) followed similar culturing media, except for using 2% FBS instead of 10% FBS. H1299 cells (ATCC) were cultured in RPMI-40 containing l-glutamine, 1% penicillin–streptomycin (1×), and 10% FBS. To form spheroids, 3000 cells were seeded in ultralow attachment 96-well plates, centrifuged to allow cell clumping, and placed in a cell culture incubator for 72 h to form cell–cell junctions. Subsequently, the spheroids were embedded in Matrigel Basement Membrane in a Mattek plate or a 24-well tissue culture plate and placed in the incubator overnight. Treatment and incubation with NNA samples were performed the following day, as the embedded spheroids began the invasion process. All cells and spheroids were maintained at 37 °C under a humidified CO_2_ atmosphere (5%).

### Confocal Uptake Studies on HeLa Cells and Spheroids

Uptake studies were performed as previously described.^23^ Briefly, HeLa cells were plated at a density of 1 × 10^4^ cells/well in a Nunc 96-well black optical plate (265300, ThermoFisher, Waltham, MA, USA) the day before the experiment. HIF-1-α ASO was labeled with a TYE563 fluorophore, and the ND scaffolds were labeled with 1% Cy5 phospholipid. ASO–ND and NNA conjugates (100 nM) were incubated with cells for 3 and 24 h, and cells were fixed with 4% paraformaldehyde. Cells were washed three times using sterile 1× PBS (pH 7.4) and stained using DAPI. For each condition involved in spheroid imaging, a minimum of 5 spheroids were embedded in Matrigel and treated with 100 nM NNA (sample 3) or ASO only and incubated for 24 h. The following day, spheroids were rinsed with sterile 1× PBS three times and replaced with fresh PBS prior to imaging. Images were acquired on a Nikon Ti2 Eclipse confocal microscope (Minato City, Tokyo, Japan) using a 60× oil objective for cells (or a 20× objective for spheroids), Nikon Elements, perfect focus, and a C2 laser scanning system. Z-stacks were collected with a 0.2 μm step size for cells and a 5 μm step size for spheroids. Images were analyzed by using ImageJ.

### SRB1 Mediated Uptake of ASO–NDs and NNAs into Cells and Spheroids

HeLa cells were plated at a density of 6 × 10^4^ cells/well in tissue-culture treated 12-well plates the day before the experiment. A minimum of 5 spheroids were embedded in Matrigel the day before the experiment. Cells and spheroids were treated with 50 μM BLT-1 in serum-free DMEM containing 0.1% BSA (v/v) for 1 h. Cells (15 nM ND scaffold, 15 nM ND) and spheroids (6 nM ND scaffold, 150 nM ASO) were treated with ASO–ND or NNA conjugates and incubated with cells for 2 h, prior to washing cells three times with 1× PBS before adding trypsin to dissociate cells from the surface or dissociate the spheroid into individual cells. The cells were collected and washed with 1× PBS two times before adding fresh PBS and collecting and analyzing the individual cells for flow cytometry assessment on a Beckman Coulter Cytoflex (Pasadena, CA, USA) to measure cell associated Cy5 fluorescence intensity. Fluorescence intensity was compared against cells not treated with BLT-1 containing ASO–ND or NNA conjugates. Histograms were prepared using FlowJo software (FlowJo LLC, Ashland, OR, USA).

### Sensitized FRET Measurements of ASO–NDs and NNAs in HeLa Cells

HeLa cells were plated at a density of 1 × 10^4^ cells/well in a Nunc 96-well black optical plate the day before the experiment. HIF-1-α ASO was labeled with a TYE563 fluorophore, and the ND scaffolds were prepared with 1% Cy5 phospholipid. ASO–ND and NNA conjugates (100 nM) were incubated with cells for 3 and 24 h, and cells were fixed with 4% paraformaldehyde. Cells were washed three times using PBS (pH 7.4) and stained using DAPI before imaging on a Nikon Eclipse Ti microscope (Minato City, Tokyo, Japan) using a 100× oil objective, reflective interference contrast microscopy (RICM), Nikon Elements, perfect focus, epifluorescence illumination, and three cubes for sensitized emission acquisition. The filter sets for the three cubes include donor channel TYE563 [ex: ET545/25x, em: ET605/70m, dichroic (T565lpxr)]; acceptor channel Cy5 [ex: ET620/60x, em: ET700/75m, dichroic (T660lpxr)]; FRET channel TYE563 [excitation ET550/40m, Cy5 emission S700/75m, dichroic (ZT647rdc)] from Chroma Technology (Bellows Falls, VT, USA). The exposure time was set at 200 ms for all channels. Control samples for FRET included ASO mixed with NNA (unconjugated), ASO only, and ND scaffold only. Image processing was performed using ImageJ and was modified as described in ref (56). Image processing was optimized to identify the Cy5 acceptor fluorescence to calculate the FRET index (Figure S4). The correction factors were calculated as shown in eqs 5 and 8 to correct for bleedthrough of donor emission into the acceptor channel and determine crosstalk. For eq 5, the values were determined with samples that contained donor only and no acceptor, whereas for eqs 6 and 8, the values were determined using acceptor only and no donor. These values were empirically calculated for our system using our specific microscope and filter setup. These factors were input into the FRET efficiency equation (eq 9) to calculate the sensitized FRET of the fluorescence pixels associated with the ASO and ND in the cells. Note that “*A*” represents the fluorescence intensity from the donor (ASO) channel, “*B*” represents the fluorescence intensity from the FRET channel, and “*C*” represents the fluorescence intensity acquired from the acceptor (Cy5 ND).ASO Crosstalk
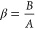
5ND Cross-Excitation
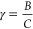
6ASO Cross-Excitation Crosstalk
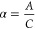
7FRET Crosstalk
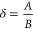
8
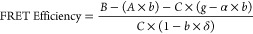
9

### RT-qPCR to Assess HIF-1-α Levels after *in Vitro* Treatment with ASO–ND or NNA Using EZN2968

HeLa, KPC, LX-2 Human Hepatic Stellate, and HepG2 cells were plated at a density of 5 × 10^4^ cells/well in tissue-culture treated 24-well plates the day before the experiment. A minimum of 5 spheroids were embedded in Matrigel and placed in 24 well plates, per condition, the day before the experiment. ASO–NDs (NDs 1–2, 4) and NNAs (NDs 3, 5) conjugated specifically to the EZN2968 ASO (Table S3), EZN2968 only, and scrambled EZN3088 on a ND scaffold were incubated with cells (100 nM) or spheroids (550 nM) for 24 h. Cells and spheroid were lysed using QIAZOL, and total RNA was collected as per the accompanying QIAGEN kit procedure. RNA was reverse transcribed to prepare cDNA in a T100 Thermal Cycler (Bio-Rad, Hercules, CA, USA). HIF-1-α mRNA levels were quantified using quantitative real time PCR (RT-qPCR) using PerfeCTa SYBR Green FastMix (using their accompanied fast 2-step cycling protocol), 50 μM custom primers (Table S3), and a Roche Lightcycler 96 (Basel, Switzerland) instrument. Relative quantification of mRNA levels was determined by using the ΔΔCt method with 18S mRNA levels for an internal control.

### MTT Assay to Assess Cell Viability

The MTT assay was performed as described previously.^23^ HeLa cells were plated at a density of 1 × 10^4^ cells/well in tissue-culture-treated 96-well plates the day before the experiment. The following day, the ASO–NDs and NNA samples (NDs 1–5) containing EZN2968, soluble EZN2968 ASO, and scrambled ASO–ND (EZN3088) were incubated with cells for 24 h. The cells were rinsed with fresh media prior to adding a 1:1 ratio (total volume = 100 μL) of phenol red-free and serum-free DMEM and prewarmed (37 °C) MTT solution. Cells were placed in an incubator at 37 °C for 3 h before adding 150 μL of prewarmed MTT solvent. Subsequently, the plate was gently agitated for 15 min before measuring the optical density at 590 nm on a plate reader. Cell viability was assessed by determining the cytotoxicity and normalizing to the untreated control set at 100%.

### Mice Acclimatization and Tail-Vein Injection of ND Scaffold, NNAs, and ASO

10-week-old male C57BL/6 mice were allowed to acclimate to their environment for 72 h before injection. Prior to injection, the mice were exposed to a heat lamp for 10 min to dilate the tail vein. Mice were restrained using an acrylic mouse restrainer, and the injection site was cleaned with 70% ethanol. ASO and NNA were injected via the tail-vein into the mice at 0.7 mg/kg, and the ND scaffold for the NNA conjugate was injected at 1 μM to best account for the concentration of the discs in the NNAs.

### Murine *in Vivo* and *ex Vivo* Fluorescence Imaging

Mouse underside hair was removed using Nair, and mice were anesthetized in a Fluovac induction chamber from Harvard Apparatus (Holliston, MA, USA) using 5% isoflurane, gradually reduced to 2%, for 5 min until they had fallen asleep. At 6 and 24 h postsample injection, the mice were imaged using the Ami HTX-Optical imager from Spectral Instruments Imaging (Tucson, AZ, USA) using 10 s exposure, 1.5 cm object height, low binning (2), 2.0 FStop, 50% excitation power, excitation of 640 nm, and emission of 710 nm. At 48 h postsample injection, the mice were sacrificed using CO_2_ and the spleen, lungs, kidneys, liver, and fat were harvested and imaged using the optical imager.

### RNA Isolation and RT-qPCR Quantification of HIF-1-α from Mice Organs

Harvested organs from the sacrificed mice were collected and rapidly purified after *ex vivo* imaging to reduce RNase exposure. Each 20 mg section of an organ was supplemented with a metal bead homogenizer and 350 μL of QIAZOL and added to a BeadBug 6 Microtube Homogenizer from Benchmark Scientific (Sayreville, NJ, USA). Samples were homogenized for 3 cycles at 2500 rpm for 30 s to facilitate the release of RNA from tissue, and samples were further purified using a benchtop mini centrifuge at 6000 rpm for 1 min to remove extracellular matrix, plasma, and blood components in the supernatant. Supernatant was diluted with a 6-fold volume of QIAZOL, and samples were purified by adding 1:1 (v/v) of QIAZOL and 70% ethanol to each sample. Organs were homogenized using a syringe to facilitate the release of RNA, and the samples were purified to isolate RNA using the QIAGEN RNA extraction kit. RNA isolation, cDNA synthesis, and RT-qPCR were performed as described above.

## Supporting Information Available

Helical wheel diagrams for ApoA1 mimetic peptides; DLS measurements of ASO–NDs and NNAs; raw fluorescence intensity from BLT-1 treatment; flowchart for calculating FRET; sensitized-FRET images for control ND and ASO samples; quantification of HIF-1-α levels for harvested organs from mice; comparing the DNA copies on gold and ND scaffolds; lipids prepared for ND scaffolds; nucleic acids used for this study (PDF)

## Abbreviations

ND: nanodisc
HDL: high density lipoprotein
HIF-1-α: hypoxible inducible factor 1 alpha
mRNA: messenger RNA
ASO: antisense oligonucleotide
NNA: nanodiscoidal nucleic acid
deoxyribozymes: DNAzymes
