# Supplementary material for: Nanodiscoidal Nucleic Acids for Gene Regulation
Source: ACS Chem Biol. 2023 Nov 1;18(11):2349–67. doi: 10.1021/acschembio.3c00038 (PMC10660333; doi:10.1021/acschembio.3c00038)
Supplement: Supplementary file 1 — cb3c00038_si_001.pdf [file cb3c00038_si_001.pdf]

Nanodiscoidal Nucleic Acids for Gene Regulation

Supporting Information

Radhika Sharma,[†] Steven Narum,[§] Shuhong Liu,[†] Yixiao Dong,[†] Kyung In Baek,[§] Hanjoong Jo,[§] and Khalid Salaita[†§]*

[†] Department of Chemistry, Emory University, Atlanta, Georgia 30332, United States

[§] Wallace H. Coulter Department of Biomedical Engineering, Georgia Institute of Technology and Emory University, Atlanta, GA, 30332, United States

* Correspondence should be addressed to Khalid Salaita: k.salaita@emory.edu

Figure S1: Helical wheel diagrams for the 22A peptide (A) and the peptides with cysteine insertion(s). 1
Figure S2: DLS measurements of ASO-NDs and NNAs before and after DNA addition. 2
Figure S3: Raw intensity values from the uptake of DNA-NDs and NNAs with and without BLT-1 treatment. 3
Figure S4: Flowchart detailing the the process for calculating FRET efficiency. 4
Figure S5: Sensitized-FRET microscopy images for control ND, ND+DNA, and DNA only samples. 5
Figure S6: H1299 3D volume Z-stack images for NNAs and ASO Only 6
Figure S7: Quantification of HIF-1-α levels after NNA treatment *in vivo*. 7
Table S1: Comparing the amount of DNA copies on ND and AuNP scaffolds. 8
Table S2: Lipids used for preparing the ND scaffolds.. 8
Table S3: Nucleic acid sequences used for this study. 8

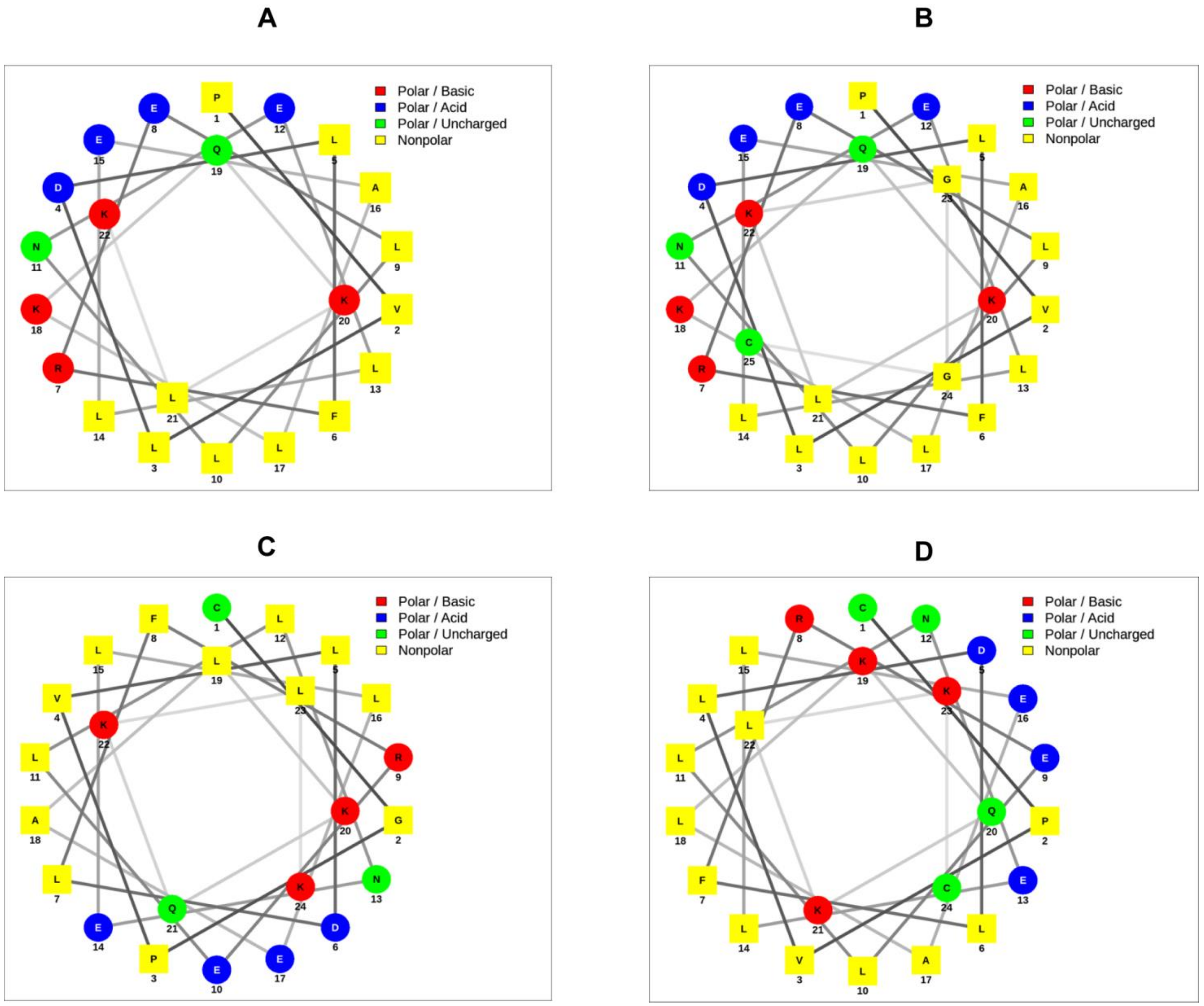

Figure S1: Helical wheel diagrams for the 22A peptide (A) and the peptides with cysteine insertion(s) for peptides B – D. Peptide sequences are listed in **Figure 1c**. Helical wheels were generated using NetWheels.

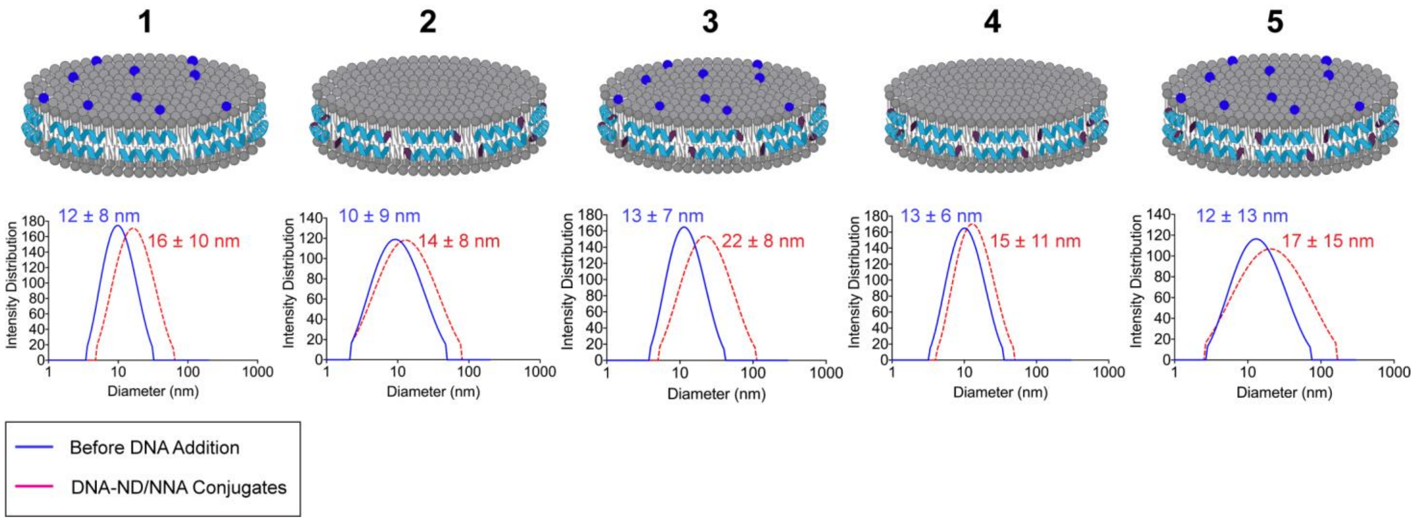

Figure S2: DLS measurements of ASO-NDs and NNAs before (blue) and after DNA addition (red). DNA coupling onto the ND scaffold shows an increase in the hydrodynamic radius. Size distribution profile is a representative graph of a typical sample (*n* = 3 independent experiments containing the ND scaffold and/or the ASO-ND or NNA conjugate).

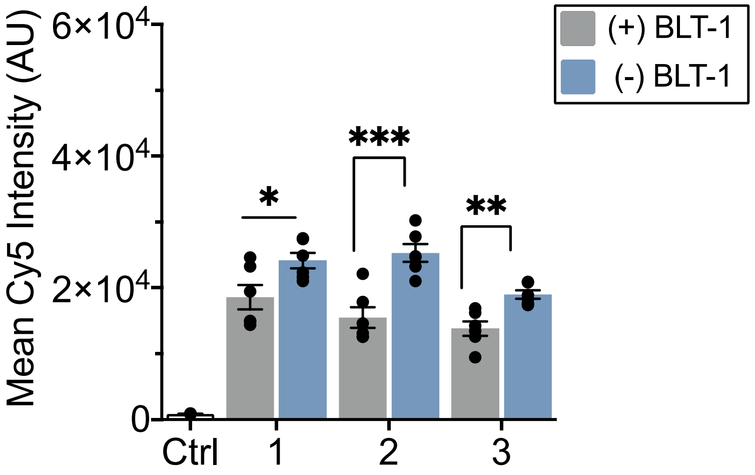

Figure S3: Raw intensity values from the uptake of DNA-NDs and NNAs with and without BLT-1 treatment. HeLa cells were incubated with 50 µm of BLT-1 to inhibit SRB1, prior to treating cells with 15 nM of the ND scaffold (400 nM ASO) for NDs 1 – 3 for 2 h before collecting cells and measuring the cell-associated Cy5 fluorescence intensity using flow cytometry. There are significant differences in uptake for each sample group treated with BLT-1 (denoted as '+') when compared to the same group with no BLT-1 treatment (denoted as '-'). This data concludes that SRB1 plays a significant role in the internalization of ASO-NDs and NNAs into cells, but other mechanisms are also involved in the uptake. Each data point represents the average from n = 6 independent replicates and * $p < 0.05$, ** $p < 0.01$, *** $p < 0.001$. Error bars represent SEM.

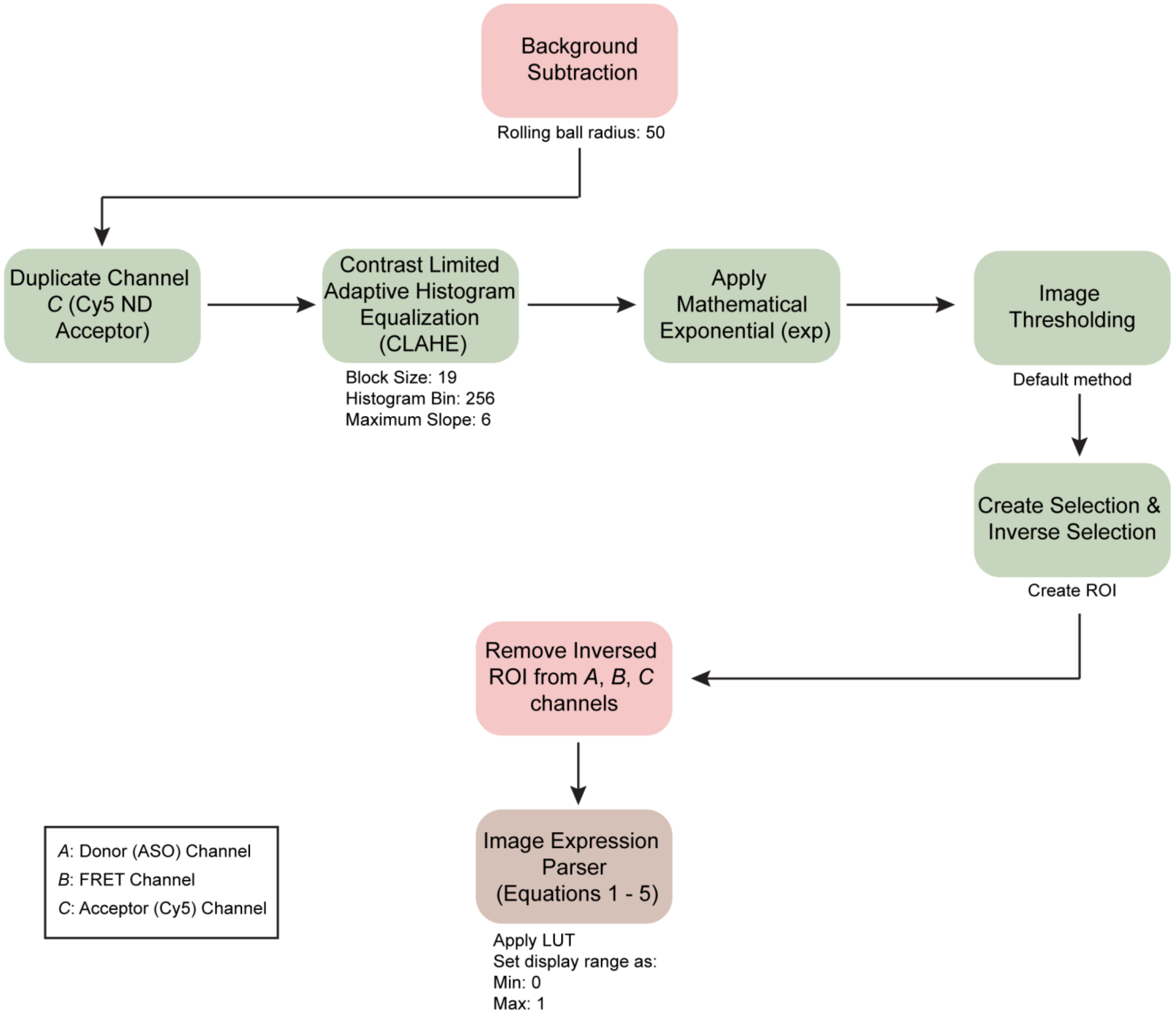

Figure S4: Flowchart detailing the image processing performed to determine FRET index (efficiency) using the Fiji/ImageJ software. Images were background subtracted using a rolling ball radius of 50 pixels for all three channels: the donor (A), FRET (B), and acceptor (C). The Cy5 acceptor (C) channel was duplicated and underwent an equalization process using the CLAHE function. CLAHE amplified "real" signal and did not amplify any signal that was contributed by noise. After CLAHE, the background on the duplicated channel was further minimized by applying a mathematical exponential before creating a threshold that enabled us to create ROI selections of regions of Cy5 intensity. Subsequently, an inverse function was applied to allow for removal of the noisy signal in the original image channels (same channel that was previously duplicated (C) and the donor (A) and FRET (B) channels included). The image was finally parsed using **Equation 9** (see **Materials and Methods**) to calculate the sensitized FRET. An LUT with a restricted image range: **0** minimum display value – **1** maximum display value.

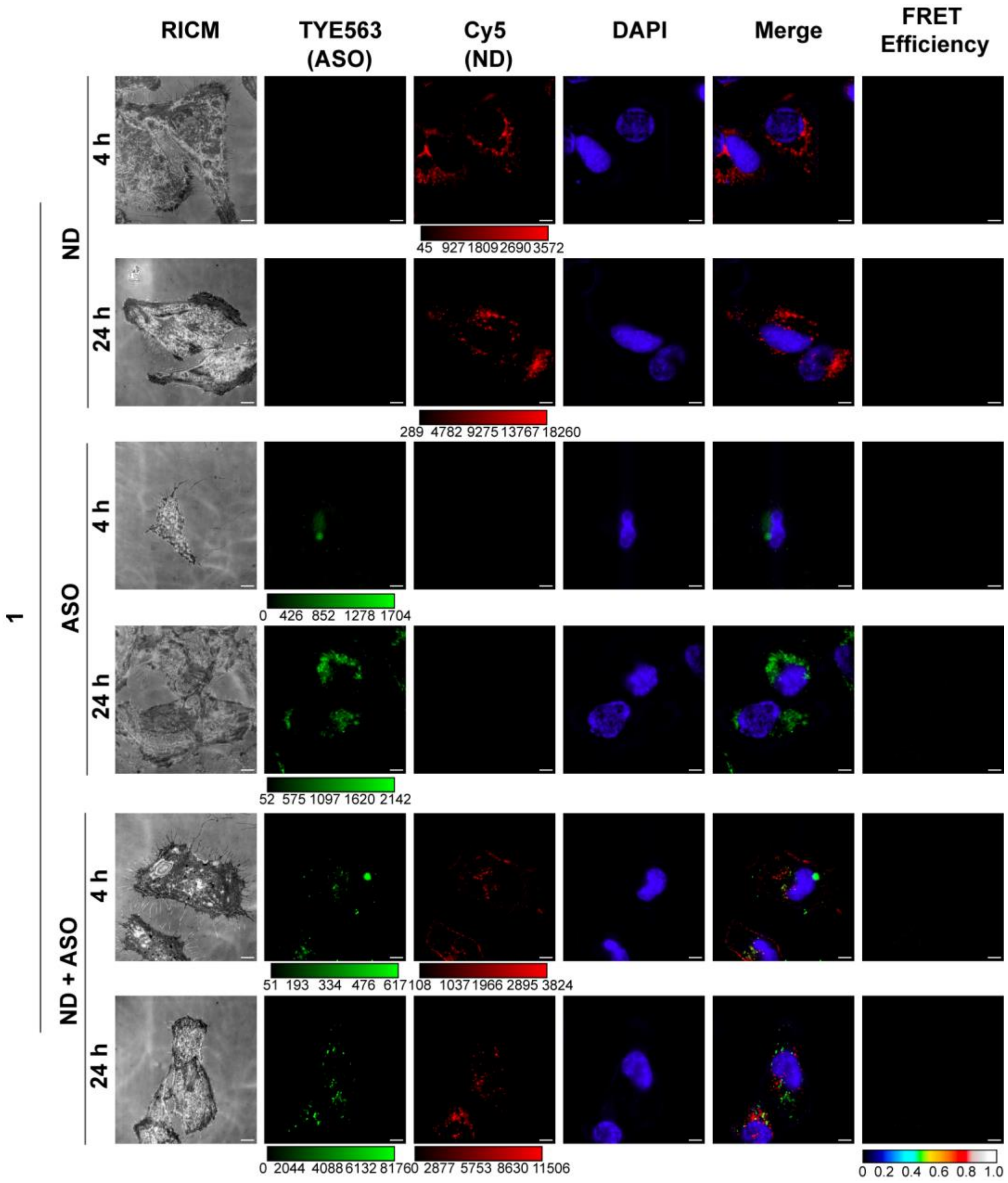

Figure S5: Representative epifluorescence images that detail the FRET efficiencies measured for the control samples for sample 1: ASO only, ND only, and ND + ASO mix but unconjugated. HeLa cells were incubated with 100 nM ASO for the ASO only and ND + ASO samples, and 6 nM of ND to best compensate for the concentration of ND in the ASO-ND for the ND only and ND + ASO sample. As described in **Figure 5**, panel includes a RICM image, and fluorescence intensity of TYE563 (ASO) and Cy5 (1% Cy5 phospholipid for NNA). Images were taken at 4 h and 24 h and cells were fixed and stained with DAPI prior to imaging with a 100× oil objective using a FRET cube. FRET images were generated from thresholding the acceptor channel (ND) and parsing the pixels and correcting for bleedthrough from donor and acceptor (ASO). Calibration bar represents FRET efficiency (set between 0 – 1.0.) As expected, the unconjugated controls do not have any FRET efficiency. Scale bar: 5 µm.

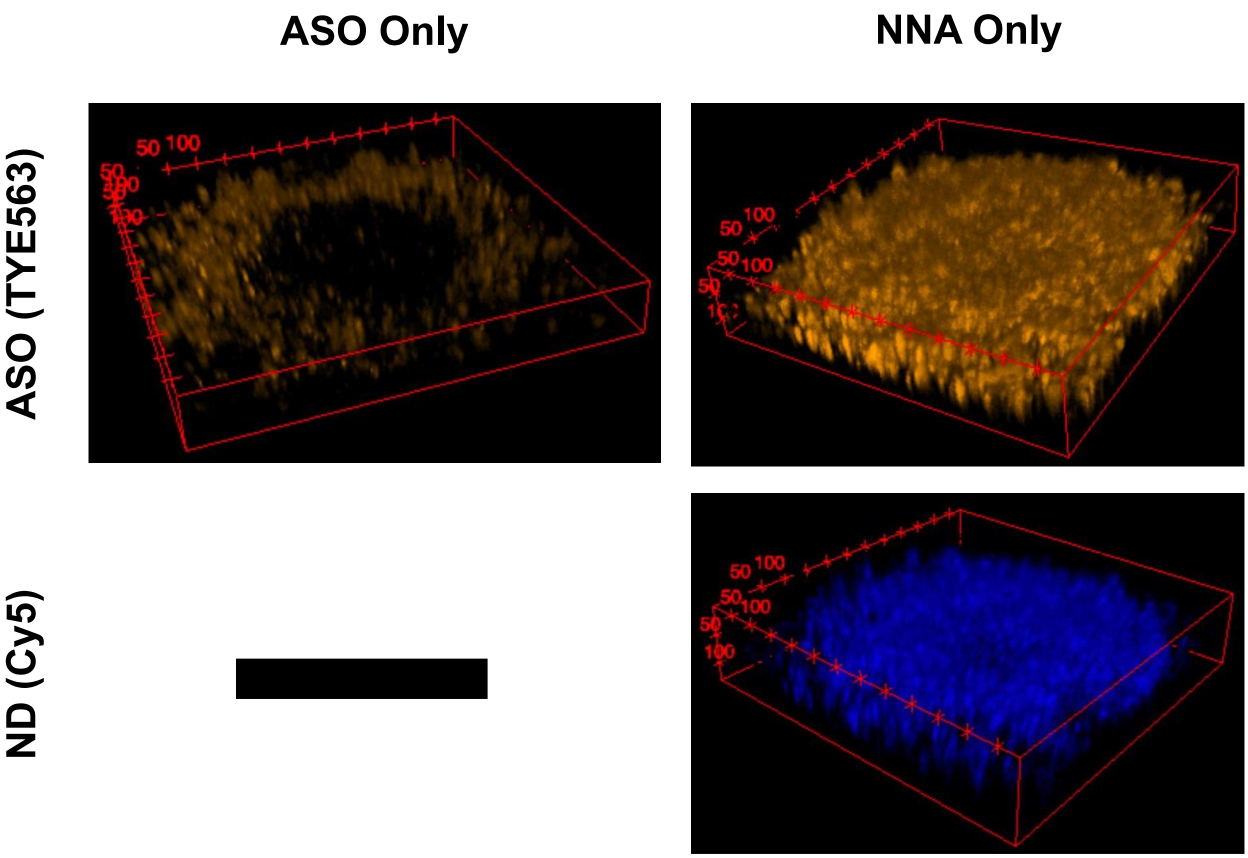

Figure S6: Z-stacks acquired from confocal microscopy for internalized NNAs and ASO (EZN2968) incubated with 3D H1299 spheroids for 24 h (ASO concentration = 100 nM). Panel includes 3D volume fluorescence images acquired from the Z-stack image slice composites for TYE563 (ASO) and Cy5 (NNA). Images detail that ASOs dosed on an ND scaffold (NNAs) are able to penetrate the core of the spheroid after 24 h of incubation whereas the ASO is not as effective in reaching the spheroid core and is mostly localized to the periphery. Note that the ASO only spheroid imaging does not have a Cy5 channel Z-stack as the spheroids were only incubated with TYE-563 containing ASO and no Cy5. Step Size: 5 µm, 20× magnification.

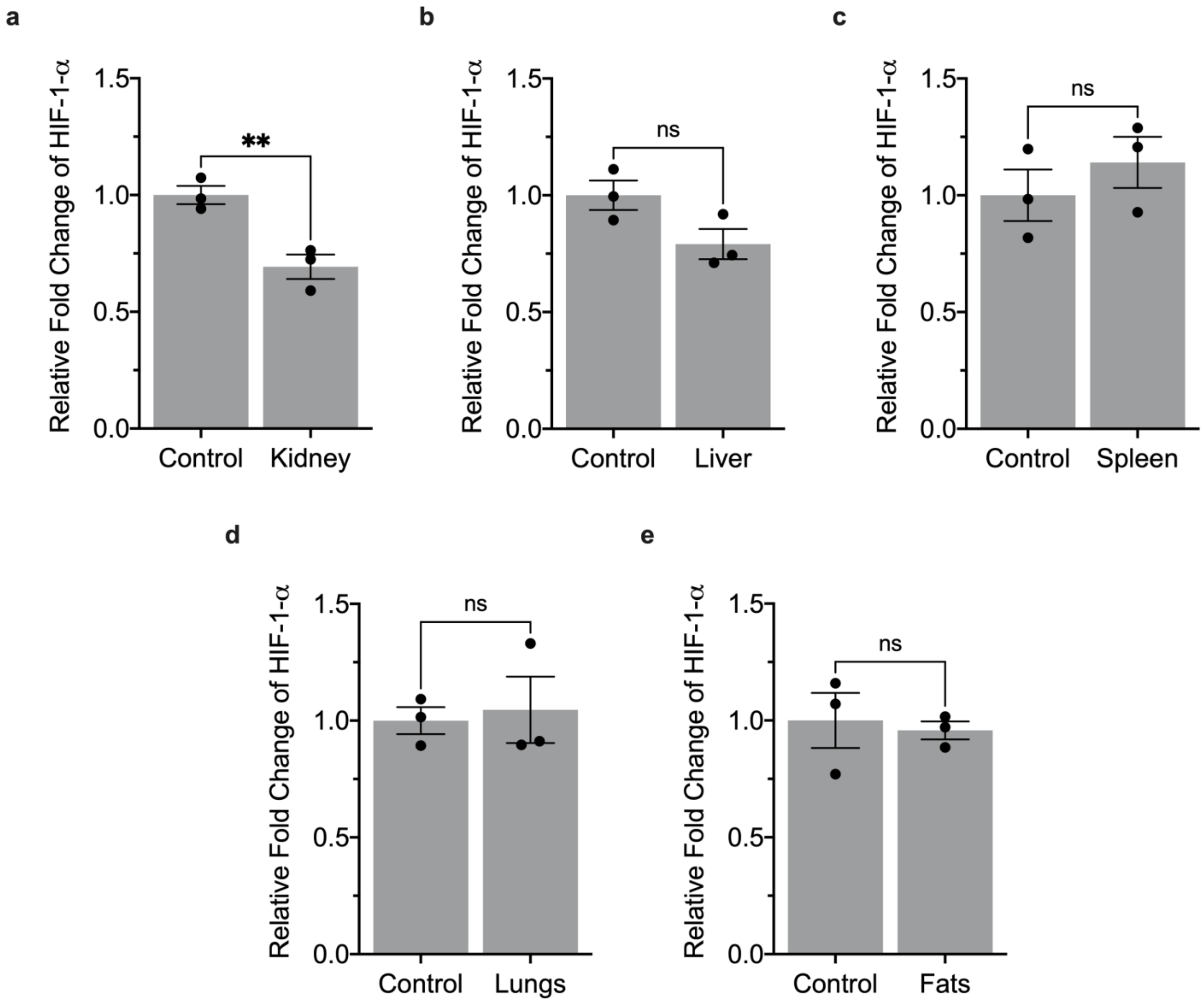

Figure S7: Quantification of HIF-1-α levels in harvested organs. ND scaffold containing no nucleic acid or NNAs (ND 3) bearing the EZN2968 anti-HIF-1-α ASO were injected into C57BL/6 mice via tail-vein injection. The ASO drug concentration was at 0.7 mg/kg whereas the ND scaffold control was injected at a concentration of 5 µM to account for the ND concentration in the NNA sample. Mice were sacrificed at 48 h post injection and organs were harvested for RNA isolation. Plots represent the quantified HIF-1-α levels for the **(a)** kidney, **(b)** liver, **(c)** spleen, **(d)** lungs, and **(e)** fats; transcript levels were normalized to identical tissues that were subject to the ND scaffold treatment without ASO as a control. Each data point represents the result from one single animal. Error bars represent SEM and * $p < 0.01$.

Table S1: Comparing the amount of DNA copies occupied per micron on gold vs. the different ND scaffolds.

Scaffold	Average DNA Copies	Surface Area (nm^2)	Footprint ($DNA/\mu m^2$)
AuNP (13 nm)	100	531	188
1	10	385	26
2	16	289	55
3	26	388	67
4	10	349	29
5	35	392	89

Table S2: Lipids used for preparing the ND scaffolds (1 – 7). Catalog number is in reference to the product as available from Avanti Polar Lipids.

Lipid	Catalog Number	Structure
DMPC (1,2-dimyristoyl-sn-glycero-3-phosphocholine)	850345	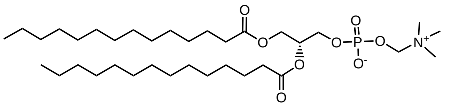
Thiol lipid (1,2-dipalmitoyl-sn-glycero-3-phosphothioethanol)	870160	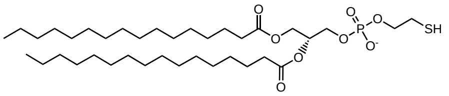
Cy5 lipid [1,2-dioleoyl-sn-glycero-3-phosphoethanolamine-N-(cyanine 5)]	810335	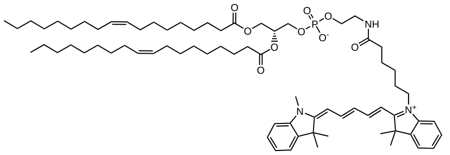

Table S3: Nucleic acid sequences used for this study. Sequences include the catalytic DNAzyme, HIF-1-α ASO sequence, scrambled ASO sequence, and primers.

"+" = LNA Modification "*" = PS Modification "3AmMo" = 3' Amino Modifier "5TYE563" = 5' TYE™ 563 Fluorophore

"rX" = RNA base "56-FAM" = 5' 6-Fluorescein fluorophore "3IABkFQ" = 3' Iowa Black® Hole FQ Quencher

ID	Sequence (5' → 3')
Catalytic Dz	ATT CCT TAA AGG CTA GCT ACA ACG ATT CTT GGC TTT
Catalytic Dz-Amine	ATT CCT TAA AGG CTA GCT ACA ACG ATT CTT GGC TTT /3AmMO/
Catalytic Dz Substrate	/56-FAM/ GCC AAG AArG rUTT AAG GAA T /3IABkFQ/
HIF-1-α ASO (EZN2968)	+T*+G*+G* C*A*A* G*C*A* T*C*C* +T*+G*+T* A
HIF-1-α ASO-Amine	+T*+G*+G* C*A*A* G*C*A* T*C*C* +T*+G*+T* A /3AmMO/
TYE563-HIF-1-α ASO-Amine	/5TYE563/ T*G*G* C*A* A* G*C*A* T*C*C* T*G*T* A /3AmMO/
Scrambled HIF-1-α ASO-Amine (EZN3088-Amine)	+C*+G*+T* C*A*G* T*A*T* G*C*G* +A*+A*+T* C /3AmMO/
HIF-1-α Forward Primer	TAT GAG CCA GAA GAA CTT TTA GGC
HIF-1-α Reverse Primer	CAC CTC TTT TGG CAA GCA TCC TG
18S Forward Primer	AGG AAT TGA CGG AAG GGC ACC A
18S Reverse Primer	GTG CAG CCC CGG ACA TCT AAG
